# Perioperative Antibiotic Prophylaxis Duration in Patients Undergoing Cystectomy With Urinary Diversion: A Randomized Clinical Trial

**DOI:** 10.1001/jamanetworkopen.2024.39382

**Published:** 2024-10-18

**Authors:** Maria C. Thurnheer, Astrid Schürmann, Markus Huber, Jonas Marschall, Patrick Y. Wuethrich, Fiona C. Burkhard

**Affiliations:** 1 Department of Infectious Diseases, Inselspital, Bern University Hospital, University of Bern, Bern, Switzerland; 2 Department of Anesthesiology and Pain Medicine, Inselspital, Bern University Hospital, University of Bern, Bern, Switzerland; 3 Division of Infectious Diseases, Washington University School of Medicine, St Louis, Missouri; 4 Department of Urology, Inselspital, Bern University Hospital, University of Bern, Bern, Switzerland

## Abstract

**Importance:**

Perioperative antibiotic prophylaxis (PAP) reduces the incidence of surgical site infections (SSIs). Guidelines recommend 24 hours or less of PAP for cystectomy with urinary diversion; however, evidence specifying optimal PAP duration for this surgery is lacking, and prolonged administration of PAP is common practice.

**Objective:**

To establish noninferiority of 24-hour PAP vs extended-duration PAP in preventing SSIs within 90 days after cystectomy with urinary diversion.

**Design, Setting, and Participants:**

This single-center, noninferiority randomized clinical trial was performed from April 18, 2018, to August 18, 2022, in patients aged older than 18 years undergoing elective open cystectomy with urinary diversion. Exclusion criteria were contraindications to administered drugs and inability to follow study procedures.

**Intervention:**

PAP administered for 24 hours (24-hour PAP group) vs PAP until all catheters and stents were removed (extended PAP group).

**Main Outcome and Measures:**

The primary end point was the rate of SSI, and the secondary end points included all-cause mortality, both within 90 days after surgery. Noninferiority of the 24-hour PAP treatment was assessed by comparing the 90% CI (corresponding to a significance level of α = .05) with the predefined noninferiority margin of 10%.

**Results:**

A total of 95 patients were randomly assigned to the 24-hour PAP group (median [IQR] age, 69.3 [63.1-76.8] years; 66 males [69.5%]) and 98 to the extended PAP group (median [IQR] age, 69.5 [60.8-75.5] years; 68 males [69.4%]). Patients in the 24-hour PAP group received PAP for a median of 1 day (IQR, 1-1 day), and patients in the extended PAP group received PAP for a median of 8 days (IQR, 7-10 days). No significant differences in SSIs occurring within 90 days were found (24-hour PAP group, 8 patients [8.4%]; extended PAP group, 12 patients [12.2%]; *P* = .53). The risk difference for 90-day cumulative SSI incidence was −3.8% (90% CI, −11.1% to 3.4%), establishing noninferiority of 24-hour PAP vs extended PAP to prevent SSI. Mortality was not significantly different between groups.

**Conclusions and Relevance:**

The findings of this randomized clinical trial demonstrate noninferiority of 24-hour PAP vs extended-duration PAP in preventing SSIs within 90 days after cystectomy with urinary diversion and may contribute to antibiotic stewardship efforts in urology.

**Trial Registration:**

ClinicalTrials.gov Identifier: NCT03305627

## Key Points

**Questions:**

How does 24 hours of perioperative antibiotic prophylaxis (PAP) compare with the commonly used prolonged PAP duration in preventing surgical site infection (SSI) in patients undergoing open cystectomy?

**Findings:**

In this randomized clinical trial, 8 of 95 patients in the 24-hour PAP group and 12 of 98 patients in the extended PAP group developed SSI within 90 days after surgery.

**Meaning:**

These findings establish noninferiority of 24-hour PAP compared with extended PAP in preventing SSI and may help to reduce unwarranted prolonged antibiotic exposure.

## Teaser

This randomized clinical trial compares 24-hour vs extended perioperative antibiotic prophylaxis to prevent surgical site infection in patients undergoing cystectomy with urinary diversion.

## Introduction

Radical cystectomy with pelvic lymph node dissection and urinary diversion is the best treatment option for patients with muscle-invasive bladder cancer. Simple cystectomy with urinary diversion is a treatment option in patients with therapy-refractory low-capacity or painful bladders. This type of surgery is associated with a high rate of postoperative complications, including surgical site infection (SSI).^1,2,3,4,5,6,7,8,9,10^ Combined with optimized perioperative care, perioperative antibiotic prophylaxis (PAP) reduces the SSI incidence by lowering the bacterial load at the site and time of intervention.^3,4,7,11,12,13,14,15,16^

Guidelines recommend PAP therapy for 24 hours or less for all clean-contaminated procedures.^16,17,18,19,20,21,22,23,24^ However, evidence specifying the optimal duration of PAP for open cystectomy with urinary diversion is lacking. While a recent meta-analysis of PAP duration^24^ included data on a variety of urologic procedures, open cystectomies were not explicitly covered.^25^ Low adherence to guideline recommendations and high interhospital variability of PAP duration have been reported in radical cystectomy, and extended (>48 hours) PAP duration is still common.^26,27^ A recent systematic review confirmed substantial heterogeneity in the choice and duration of PAP in patients undergoing radical cystectomy^6^ and showed no clear benefit associated with extended PAP duration. Unwarranted prolonged use of antibiotics may incur high costs, trigger adverse drug reactions, drive the emergence of bacterial resistance, and lead to an increasing number of difficult-to-treat infections.^13,28,29,30,31^

To date, 2 small, prospective, nonrandomized studies have shown efficacy of 24-hour compared with 72-hour PAP treatment in preventing postoperative infections in patients undergoing radical cystectomy.^5,32^ Larger randomized clinical trials supporting these findings and the current guideline recommendations are lacking. The primary objective of the current study was to investigate whether the recommended 24 hours or less of PAP is noninferior to the traditionally used extended-duration PAP (>48 hours, until removal of all catheters and stents) in preventing SSI within 90 days after surgery in patients undergoing open cystectomy and urinary diversion at a single high-caseload center.

## Methods

### Study Design

This prospective, unmasked, noninferiority randomized clinical trial was conducted from April 18, 2018, to August 18, 2022, at the Department of Urology, University Hospital Bern. The trial was approved by the Swiss government’s local ethics committee and conducted in compliance with the principles of the Declaration of Helsinki^33^ and Good Clinical Practice.^34^ The full study protocol is provided in Supplement 1. All patients gave preoperative written informed consent to participate. The study follows the Consolidated Standards of Reporting Trials (CONSORT) reporting guideline.

During the study period, patients scheduled for open cystectomy for oncologic or functional indications at the study center were screened for study eligibility during the preoperative assessment or, at the latest, the day before surgery by a member of the study team. Patients older than 18 years with planned open cystectomy with urinary diversion were included. Patients with contraindications to the class of drugs used, who were pregnant or breastfeeding, and who were unable to follow study procedures were excluded. After giving informed consent, patients were randomly assigned to receive PAP for either 24 hours (24-hour PAP group [intervention]) or until all ureteral catheters and stents were removed (extended PAP group [current practice, duration not standardized]). Randomization was performed by computer-based concealed block randomization, stratified for age, sex, and body mass index (BMI; calculated as weight in kilograms divided by height in meters squared), and equal proportions were assigned to the 2 study groups.

### End Points

The primary end point was 90-day cumulative incidence of any SSI. Secondary end points were 90-day postoperative mortality, rates of adverse events (any untoward medical occurrence [clinical or laboratory event] and antibiotic-related adverse events [nephrotoxicity, hematotoxicity, hepatotoxicity, gastrointestinal side effects, hypersensitivity reactions, *Clostridium difficile* colitis, or resistant or multiresistant bacteria in subsequent urine samples]), additional antibiotic prescription days during hospitalization for cystectomy, hospital length of stay, and both asymptomatic bacteriuria and febrile urinary tract infection (UTI). Surgical site infections and UTIs were assessed within 90 days after surgery, diagnosed by the treating urologist based on clinical and laboratory information, and classified and recorded as defined in the literature.^35,36^ All suspected and diagnosed SSIs were verified and validated based on predefined criteria by an infectious disease specialist (M.C.T.) and a physician not involved in the clinical treatment (A.S.) (eTable 3 in Supplement 2). Of note, typical UTI symptoms are lacking after cystectomy; therefore, febrile UTI was defined as bacteriuria plus fever in the absence of an alternative infection focus. A 90-day observation period was used to account for potentially delayed SSI onset when administering prolonged PAP and to allow benchmarking with other SSI surveillance data tracking infections for 90 days.

### Time Course and Intervention

Patients in the 24-hour PAP group received PAP for 24 hours following incision, while patients in the extended PAP group received antibiotics until all catheters and stents were removed (>48 hours). Antibiotic combinations reflected local standard practice (details provided in eTables 1 and 2 in Supplement 2). All patients with orthotopic neobladder reconstruction received antibiotic prophylaxis at the time of transurethral catheter removal, irrespective of PAP duration (eTable 1 in Supplement 2). Patients remained hospitalized until all catheters were removed and they were considered competent in managing the urinary diversion (local standard practice).

Data were collected at baseline, on the day of surgery, and on postoperative days 1, 2, 3, 5, 7, 10, 30 (±7 days), and 90 (±10 days). Clinical and laboratory data including urine analysis and urine culture, time point of diagnosis and management of SSI, and asymptomatic bacteriuria and UTI were recorded using an electronic clinical record form. Each episode of SSI was reviewed and assessed in detail by an infectious disease specialist (M.C.T.) and a physician not involved in the clinical treatment (A.S.) according to predefined criteria (eTable 3 in Supplement 2). Asymptomatic bacteriuria and UTI were considered 1 episode if the diagnosis remained the same and the time between recordings was 3 days or less. Adverse events and study exclusion were recorded for each eligible patient. Duration of antibiotic treatment was calculated based on prescription records available for the index hospitalization during which open cystectomy was performed. Rates of antibiotic prescriptions were calculated per group as antibiotic days divided by hospitalization days.

### Determination of Sample Size

Based on a study by Hara et al^5^ and on available information on local SSI incidence after open cystectomy, we assumed a lower SSI rate in the 24-hour PAP group than in the extended PAP group, with SSI incidence of 10.0% and 13.5%, respectively. For hypothesis testing and study design, we adopted a noninferiority design in which we considered 24-hour PAP treatment as noninferior to extended PAP treatment within a clinically acceptable margin of 10% with regard to study end points. Assuming a significance level of α = .05, power of 80%, and dropout rate of 40%, we derived a required sample size of 198 patients such that 99 were included in each treatment group. Based on a mean frequency of 79 open cystectomies performed annually (2010-2015), we projected a study duration of 3 years. The study period had to be extended due to COVID-19–related hospital policies that restricted both research activities and surgical interventions during the early phase of the pandemic.

### Statistical Analysis

For descriptive measures, categorical variables, including age group, sex, self-reported race and ethnicity (White and other [Black or Hispanic] in order to identify potential racial or ethnic bias), BMI range, diagnosis of muscle-invasive bladder cancer, comorbidities, medications, and surgical procedures, were summarized using counts and percentages. Numerical variables with mean (SD) were used in cases of normally distributed variables and with median (IQR) otherwise. The balance of baseline characteristics in the 2 treatment groups was assessed using standardized mean differences. For statistical inference, group comparisons were based on the χ^2^ test for categorical outcomes, Student *t* test for normally distributed quantitative outcomes, and unpaired 2-sample Wilcoxon test otherwise. A significance level of α = .05 was used throughout the study.

**Figure 1.**
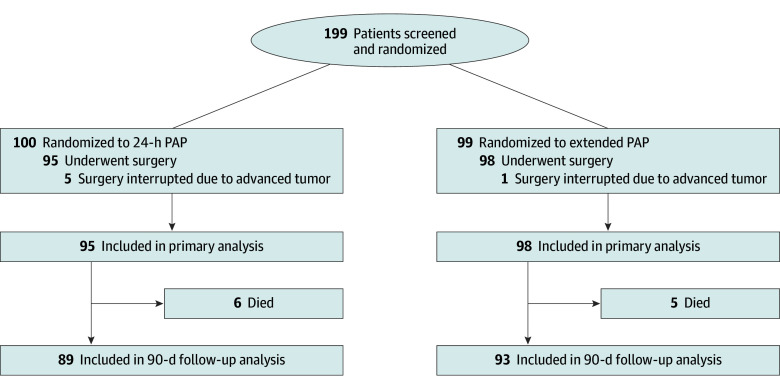
Flow Diagram of Study Patients PAP indicates perioperative antibiotic prophylaxis.

The primary end point of the risk difference for 90-day cumulative SSI incidence between the 2 treatment groups was computed using a competing-risk survival, per-protocol analysis with the 2 possible events of SSI and death. The per-protocol analysis included all randomized patients except 6 for whom surgery was interrupted due to advanced tumor (Figure 1). As a sensitivity analysis, we performed 2 intention-to-treat analyses in which the missing outcomes of the 6 excluded patients were imputed with (1) an SSI at day 10 and no deaths occurring and (2) no SSI over the course of the follow-up and no deaths occurring. The results of this sensitivity analysis are provided in eTable 4 in Supplement 2.

**Figure 2.**
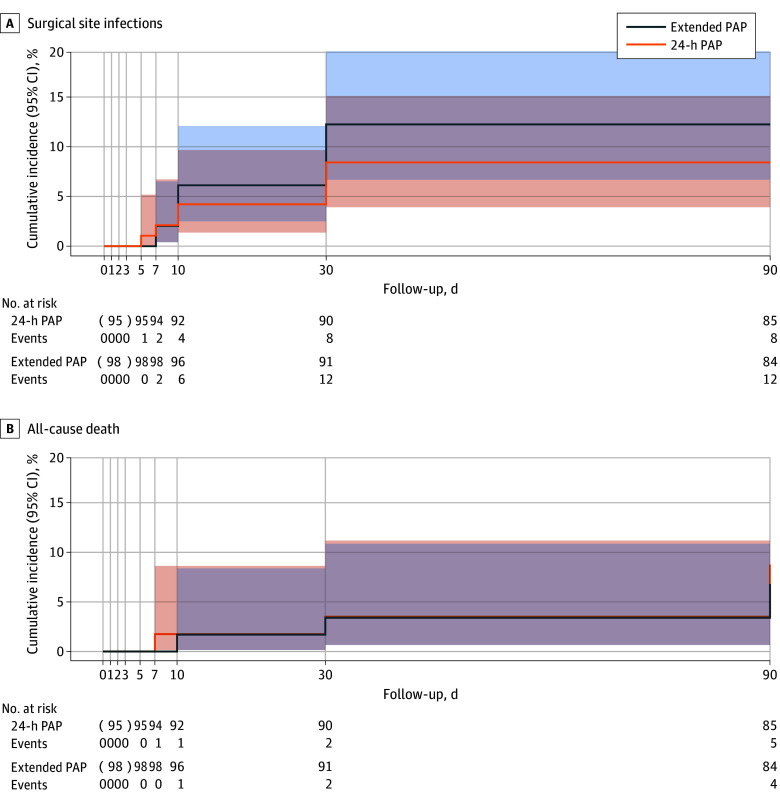
Competing-Risk Analysis for Surgical Site Infections and Death A tabular presentation of cumulative incidence data is provided in eTable 5 in Supplement 2. PAP indicates perioperative antibiotic prophylaxis.

The Gray nonparametric test was used to compare the cumulative incidence functions. Hazard ratios were computed using a competing-risk regression. Noninferiority of the 24-hour PAP treatment was assessed by comparing the 90% CI (corresponding to a significance level of α = .05) with the predefined noninferiority margin of 10.0%. Rates of asymptomatic bacteriuria, UTI, and both indications were calculated and compared as previously mentioned. Data capture and randomization were performed using REDCap^37,38^ (Vanderbilt University), while Stata MP, version 16.1 (StataCorp LLC) and R, version 4.0.2 (R Foundation) were used for statistical analysis and creation of Figure 2. The R package tidycmprsk was used for the competing-risk analysis.^39^

## Results

### Patient Demographics and Clinical Presentation

During the study period, a total of 249 patients underwent open cystectomies at the study center, of whom 199 were screened and randomized (Figure 1), resulting in 100 patients allocated to the 24-hour PAP group and 99 to the extended PAP group. Five patients in the 24-hour PAP group and 1 in the extended PAP group had advanced tumor progression discovered during surgery, and their index operation was not completed as planned. These individuals were excluded from follow-up and analysis. The study population included in the analysis therefore comprised 95 patients in the 24-hour PAP group (median [IQR] age, 69.3 [63.1-76.8] years; 29 females [30.5%] and 66 males [69.5%]; and 95 of White race [100.0%]) and 98 in the extended PAP group (median [IQR] age, 69.5 [60.8-75.5] years; 30 females [30.6%] and 68 males [69.4%]; and 96 [98.0%] of White and 2 [2.0%] of other race and ethnicity). Six deaths occurred in the 24-hour PAP group and 5 in the extended PAP group. Follow-up at postoperative day 90 was completed in all 89 (24-hour PAP) and 93 (extended PAP) surviving patients.

The study groups were stratified for sex, age, and BMI (median, 26.5 [IQR, 23.1-28.6] and 25.9 [IQR, 22.7-28.7] in the 24-hour PAP and extended PAP groups, respectively) (Table 1). Most patients were diagnosed with muscle-invasive bladder cancer (24-hour PAP arm, 80 [84.2%]; extended PAP arm, 88 [89.8%]). Prevalence of baseline comorbidities was similar in both groups.

**Table 1.** Baseline Characteristics of the Study Population

Characteristic	No. of patients (%)	
	24-h PAP (n = 95)	Extended PAP (n = 98)
Age at enrollment, median (IQR), y	69.3 (63.1-76.8)	69.5 (60.8-75.5)
Age group, y		
20-59	19 (20.0)	19 (19.4)
60-80	63 (66.3)	70 (71.4)
>80	13 (13.7)	9 (9.2)
Sex		
Female	29 (30.5)	30 (30.6)
Male	66 (69.5)	68 (69.4)
Race and ethnicity		
White	95 (100)	96 (98.0)
Other^a^	0	2 (2.0)
BMI, median (IQR)	26.5 (23.1-28.6)	25.9 (22.7-28.7)
BMI range		
<18.5 (Underweight)	0	3 (3.1)
18.5-24.9	37 (38.9)	38 (38.8)
25-29.9	43 (45.3)	41 (41.8)
≥30 (Obesity)	15 (15.8)	16 (16.3)
Diagnosis of muscle-invasive bladder cancer	80 (84.2)	88 (89.8)
Comorbidities and medical conditions		
Diabetes	11 (11.6)	9 (9.2)
Hypertension	36 (37.9)	35 (35.7)
Kidney disease	16 (16.8)	11 (11.2)
Heart disease	21 (22.3)	20 (21.1)
Liver disease	3 (3.19)	3 (3.12)
Smoking	23 (24.7)	37 (38.1)
Previous urinary tract infection	29 (30.5)	24 (24.7)
Known colonization with multidrug-resistant microbes	2 (2.1)	2 (2.1)
Antibiotics within the past 3 mo	54 (56.8)	56 (57.7)
Immunosuppressive drugs	3 (3.3)	3 (3.1)
Neoadjuvant chemotherapy within past 3 mo	24 (26.4)	28 (29.5)
Currently receiving chemotherapy	2 (8.3)	2 (7.1)
Antihypertensives	39 (42.4)	39 (40.2)
Antidiabetics	9 (9.8)	7 (7.2)
Performed surgery		
Pelvic lymph node dissection	74 (77.9)	76 (77.6)
Open cystectomy	95 (100)	98 (100)
Performed urinary diversion		
Orthotopic ileal bladder substitute	36 (37.9)	32 (32.7)
Ileal conduit	50 (52.6)	61 (62.2)
Ileal continent catheterizable pouch	3 (3.2)	2 (2.0)
Ureterocutaneostomy	6 (6.3)	3 (3.1)
Catheter dwell time, median (IQR), d		
Ureteral stent	7 (7-10)	8 (7-10)
Urethral catheter	12 (10-16)	12 (10-32)
Central line, median (IQR), d	7 (5-7)	7 (7-10)

Abbreviations: BMI, body mass index (calculated as weight in kilograms divided by height in meters squared); PAP, perioperative antibiotic prophylaxis.

^a^ Other race and ethnicity was categorized as Black or Hispanic.

### SSIs

The PAP regimen was administered for a median of 1 day (IQR, 1-1 day) to patients in the 24-hour PAP group and a median of 8 days (IQR, 7-10 days) to patients in the extended PAP group (Table 2). A total of 20 of 193 patients (10.4%) were diagnosed with an SSI for an absolute rate of 8.4% (8 of 95 patients) in the 24-hour PAP group and 12.2% (12 of 98 patients) in the extended PAP group (*P* = .53) (Table 2).

**Table 2.** SSIs and Death

Variable	24-h PAP (n = 95)	Extended PAP (n = 98)	***P* value**
**Summary measures**			
Any SSI during follow-up, No. (%; 95% CI)	8 (8.4; 95% CI, 3.7-15.9)	12 (12.2; 95% CI, 6.5-20.4)	.53
Type of SSI, No. (%)			
Superficial	5 (5.3)	4 (4.1)	.43
Deep	2 (2.1)	5 (5.1)	
Organ or space	1 (1.1)	3 (3.1)	
Time after surgery to SSI, median (IQR), d	11 (8-22)	14 (8-23)	.76
PAP duration, median (IQR), d	1 (1-1)	8 (7-10)	<.001
Length of hospital stay, median (IQR), d	15 (12-17)	15 (12-17)	.40
Length of hospital stay with SSI, median (IQR), d	19 (17-30)	22 (14-30)	.87
Death during follow-up, No. (%; 95% CI)	6 (6.3; 95% CI, 2.4-13.2)	5 (5.1; 95% CI, 1.7-11.5)	.96
Cause of death, No. (%)			
Unclear, after discharge	1 (11.7)	3 (60.0)	NA
Respiratory failure and/or cardiac arrest	2 (33.3)	1 (20.0)	
Tumor progression	3 (50.0)	1 (20.0)	
**Competing-risk regression, HR (95% CI)**			
SSI^a^	0.68 (0.28-1.64)	1 [Reference]	.39
Death	1.29 (0.35-4.75)	1 [Reference]	.70

Abbreviations: HR, hazard ratio; NA, not applicable; PAP, perioperative antibiotic prophylaxis; SSI, surgical site infection.

^a^ For the primary outcome of SSI, the risk difference (90-day cumulative incidence) between 24-hour PAP and extended PAP was −3.8% (90% CI, −11.1% to 3.4%), establishing noninferiority at a margin of 10.0%.

Competing-risk regression analysis showed a hazard ratio of 0.68 (95% CI, 0.28-1.64; *P* = .39) for SSI in the 24-hour PAP group vs the extended PAP group (Figure 2A; eTable 5 in Supplement 2). The risk difference for 90-day cumulative SSI incidence (24-hour PAP minus extended PAP) was −3.8% (90% CI, −11.1% to 3.4%), establishing noninferiority of 24-hour PAP vs extended PAP to prevent SSIs with respect to the predefined noninferiority margin of 10.0% (Table 2).

Statistically, clinical SSI manifestations were not different between the 2 groups. In the 95 patients receiving 24-hour PAP vs 98 patients receiving extended PAP, superficial SSI was diagnosed in 5 (5.3%) vs 4 (4.1%), deep SSI in 2 (2.1%) vs 5 (5.1%), and organ or space infection in 1 (1.1%) vs 3 (3.1%), respectively (*P* = .43) (Table 2). All SSIs were resolved with appropriate treatment, and no deaths directly associated with SSIs were observed.

There was no significant difference in time to SSI diagnosis. Patients remained hospitalized until they were competent in managing the urinary diversion and all catheters and stents were removed; therefore, hospital length of stay was similar for both groups (median, 15 days; IQR, 12-17 days). Patients with SSIs were hospitalized for a longer time in both groups with no difference in length of stay between the 2 groups (24-hour PAP: median, 19 days [IQR, 17-30 days]; extended PAP: median, 22 days [IQR, 14-30 days]; *P* = .87).

During the study period, a total of 11 deaths occurred. Six of 95 (6.3%) patients in the 24-hour PAP group and 5 of 98 (5.1%) patients in the extended PAP group died from causes unrelated to SSI or their index surgery (Table 2; Figure 2B; eTable 5 in Supplement 2).

### Asymptomatic Bacteriuria and Febrile UTIs

In the 24-hour PAP group, 207 episodes of asymptomatic bacteriuria were found in 817 performed urine analyses compared with 134 episodes in 828 urine analyses in the extended PAP group (rate, 25.3% vs 16.2%, respectively, *P* < .001) (Table 3). The time after surgery until the first episode of asymptomatic bacteriuria was not statistically different in the 2 groups. All asymptomatic bacteriuria episodes occurred after completion of PAP in the 24-hour PAP group, while 52 of 134 asymptomatic bacteriuria episodes (38.8%) occurred in patients still receiving PAP in the extended PAP group. Although not specified or recommended by the protocol, treatments for asymptomatic bacteriuria were prescribed after completion of PAP (per treating physician decision). Treatment rates for asymptomatic bacteriuria diagnosed after completion of PAP were similar in both groups (treatment rates, 54 of 207 (26.1%) and 19 of 82 (23.2%) in the 24-hour PAP and extended PAP groups, respectively; *P* = .67).

**Table 3.** Asymptomatic Bacteriuria, Urinary Tract Infections, Antibiotic Prescriptions, and Adverse Events

Variable	No. (%)		*P* value
	24-h PAP (n = 95)	Extended PAP (n = 98)	
**Asymptomatic bacteriuria** ^a^			
Total urine diagnostics (urine status and/or urine culture), No.	817	828	.36
Episodes	207 (25.3)	134 (16.2)	<.001
Treated	54 (26.1)	19 (14.2)	.01
Episodes after completion of PAP	207 (100)	82 (61.2)	<.001
Treated	54 (26.1)	19 (23.2)	.67
Time to diagnosis of first episode, median (IQR), d	7 (2-10)	10 (2-30)	.53
**≥1 Febrile UTI**			
Total	19 (20.0)	13 (13.3)	.21
Time to diagnosis, median (IQR), d	27 (10-42)	22 (13-29)	.83
**Antibiotics during index hospitalization for cystectomy^b^**			
Antibiotics other than PAP duration, median (IQR), d	3 (0-7)	1 (0-7)	.051
Total antibiotic d, median (IQR)	4 (1-8)	10 (8-14)	<.001
Total antibiotic d if SSI, median (IQR)	10 (6-12)	17 (9-22)	.53
Indications for treatment during index hospitalization			
Catheter removal or additional surgery	37 (38.9)	29 (29.6)	NA
Asymptomatic bacteriuria	22 (21.1)	7 (7.1)	
UTI	7 (7.4)	1 (1.0)	
Bacteremia	5 (5.3)	3 (3.1)	
SSI	5 (5.3)	6 (6.1)	
Empirical treatment	8 (8.4)	4 (4.1)	
Pneumonia	3 (3.2)	2 (2.0)	
Other^b^	2 (2.1)	0	
**Adverse events**			
Patients with adverse events, No. (%)	44 (46.3)	40 (40.8)	.56
Total adverse events, No.	52	55	.14
Adverse events related to antibiotics, No. (%)			
Rash or gastrointestinal complaints^c^	0	6 (10.9)	NA
Adverse events related to surgery, No. (%)			
Ileus	10 (19.2)	7 (12.7)	NA
Lymphocele, not infected	3 (5.8)	2 (3.6)	
Wound dehiscence, not infected	2 (3.8)	1 (1.8)	
Other surgical complications^d^	2 (3.8)	8 (14.5)	
Other adverse events			
Diarrhea not related to antibiotics	0	2 (3.6)	NA
Impaired kidney function (GFR <60 mL/min)	8 (15.4)	7 (12.7)	
Tumor progression	3 (5.8)	2 (3.6)	
Other adverse events^e^	10 (19.2)	5 (9.1)	

Abbreviations: GFR, glomerular filtration rate; NA, not applicable; PAP, perioperative antibiotic prophylaxis; SSI, surgical site infection; UTI, urinary tract infection.

^a^ Multiple episodes per patient possible.

^b^ Other indications included skin infection and unclear or not stated.

^c^ Included 5 nonspecific complaints and 1 episode of mild *Clostridium difficile* colitis, with no adverse events observed.

^d^ Other complications included accidental perforation of intestinal tract (3 patients), additional surgery not related to index surgery (4 patients), hemorrhagia (1 patient), urinoma (1 patient), and widening of ureteral anastomosis (1 patient).

^e^ Other adverse events included pain (3 patients); pancreatitis (1 patient); pancytopenia after chemotherapy (1 patient); peripheral pulmonary embolism, not life threatening (2 patients); decubitus ulcer (2 patients); dyselectrolytemia (2 patients); psychiatric problems (1 patient); discharge around percutaneous endoscopic gastrostomy tube (1 patient); prolonged leakage from surgical wound, not classified or treated as SSI (1 patient); and positive culture findings from central line tip, no signs of catheter-associated infection (1 patient).

In the 24-hour PAP group, 19 of 95 (20.0%) patients developed at least 1 episode of febrile UTI compared with 13 of 98 (13.3%) patients in the extended PAP group (*P* = .21). Time from index surgery until diagnosis of first febrile UTI did not differ between the 2 groups (Table 3).

### Prescription of Additional Antibiotics

Prescription of antibiotics for indications other than perioperative prophylaxis for the index surgery was frequent during the index hospitalization. Patients in the 24-hour PAP group received antibiotics for a median 3 additional days (IQR, 0-7 days) vs 1 day (IQR, 0-7 days) in the extended PAP group. Nonetheless, the total number of days with antibiotic treatment for any indication was higher in the extended PAP group (median, 10 days [IQR, 8-14 days] vs 4 days [IQR, 1-8 days] in the 24-hour PAP group; *P* < .001) (Table 3).

### Adverse Events

The rate of adverse events was similar between the groups (44 patients [46.3%] and 40 patients [40.8%] in the 24-hour PAP and extended PAP groups, respectively). Six of 55 (10.9%) adverse events were directly related to antibiotics in the extended PAP group, while no antibiotic-related adverse events were observed in the 24-hour PAP group (Table 3).

## Discussion

In this prospective, noninferiority randomized clinical trial, we examined the effect of 24-hour PAP compared with extended PAP on the incidence and rate of SSIs after open cystectomy and ileal urinary diversion. Our findings suggest no significant difference between the 2 PAP durations given that the prespecified noninferiority criteria were met. These findings provide needed clinical evidence to support 24-hour PAP for the prevention of SSI after open cystectomy and urinary diversion.

The demographics of the studied population were comparable with cohorts presented in the literature, and the observed overall SSI rate of 10.4% was similar or lower to previously reported findings.^2,4,5,6,12^ We also found that prolonged PAP did not reduce the rate or severity of SSIs and, therefore, provided no marginal benefit over 24-hour PAP. Importantly, there was no significant difference in crude mortality between the 2 groups within 90 days after surgery.

In our setting, the duration of hospitalization after cystectomy reflected local practice and was determined by the timing of catheter removal and delivery of instructions to patients on how to competently manage the urinary diversion. We observed no difference in length of hospital stay between the 2 groups.

We observed a higher frequency of asymptomatic bacteriuria in the 24-hour PAP group; however, this did not translate to a statistically significant difference in febrile UTIs. The rate of patients with at least 1 symptomatic UTI (19 of 95 [20.0%] in the 24-hour PAP group and 13 of 98 (13.3%) in the extended PAP group) was lower than the 69 UTI episodes in 165 patients (41.8%) reported by Ross et al^4^ in a comparable population and similar to the rate of 19.4% febrile UTIs in 217 patients after radical cystectomy reported by Haider et al^40^ in a retrospective multicenter case series. Similar to our findings, Haider et al^40^ found no association between the duration of PAP and risk for UTI.

Our study protocol allowed for the prescription of antibiotics for indications other than PAP, such as intercurrent infections or prophylaxis for catheter removal or additional surgery. Treatment of asymptomatic bacteriuria is generally not recommended; however, clinical assessment can be challenging in patients with urinary diversion as they lack typical UTI symptoms. The observed high rate of antibiotic prescriptions for asymptomatic bacteriuria in the study population may be a center-specific phenomenon. Nonetheless, it points to an area of management uncertainty that might be amenable to interventions aimed at reducing unwarranted antibiotic prescriptions.

Finally, although patients in the 24-hour PAP group received antibiotics for an additional 3 days compared with 1 additional antibiotic treatment day in the extended PAP group, total antibiotic days during the index hospitalization were higher in the extended PAP group (9.5 days vs 4.0 days in the 24-hour PAP group), demonstrating that shortened PAP duration may contribute to antimicrobial stewardship efforts.

### Limitations

This study has some limitations. It was performed in a single center and may not reflect clinical practice in different settings. The study was not designed to examine the drug combinations used for PAP, so we could not assess whether a narrower antimicrobial spectrum may have been as effective as the currently used combination. The study design was unmasked, and the initial diagnosis of suspected SSI was based on clinical assessment by the treating medical team. To ensure adherence to predefined diagnostic criteria and to mitigate subjective interpretation, any suspected SSI diagnosis was verified by an infectious disease specialist and a clinician not directly involved in patient care.

## Conclusions

To our knowledge, this randomized clinical trial is the first study to provide high-quality evidence that 24-hour PAP is noninferior to extended PAP in preventing SSI within 90 days after cystectomy and urinary diversion. This evidence may contribute to antibiotic stewardship efforts in urology.

## Notes

Supplement 1.
Trial Protocol

Supplement 2. **eTable 1.** Antibiotic Regimen Used for Perioperative Prophylaxis **eTable 2.** Perioperative Care **eTable 3.** Diagnosis of Surgical Site Infections (e CRF Form) **eTable 4.** Intention-to-Treat (ITT) Sensitivity Analysis **eTable 5.** Cumulative Incidences for Surgical Site Infection and Death

Supplement 3.
Data Sharing Statement
