# Supplementary material for: Perioperative Antibiotic Prophylaxis Duration in Patients Undergoing Cystectomy With Urinary Diversion: A Randomized Clinical Trial
Source: JAMA Netw Open. 2024 Oct 18;7(10):e2439382. doi: 10.1001/jamanetworkopen.2024.39382 (PMC11581670; doi:10.1001/jamanetworkopen.2024.39382)
Supplement: Supplement 1. — Trial Protocol [file jamanetwopen-e2439382-s001.pdf]

Clinical Study Protocol

Optimized Perioperative Antibiotic Prophylaxis in Radical cystectomy (PAPRAC)

Study Type:	Prospective randomized controlled clinical non-inferiority trial
Study Categorisation:	A
Study Registration:	Registration number (from FOPH portal) eventually other registries and numbers if applicable
Study Identifier:	2017-01480
Sponsor, Sponsor-Investigator or Principal Investigator:	Dr.med. M.C. Thurnheer<br>Department of Infectious Diseases<br>University Hospital Bern<br>3010 Bern<br>christine.thurnheer@insel.ch<br>Tel: 031 632 99 92
Investigational Product:	Duration of perioperative antibiotic prophylaxis in radical cystectomy
Protocol Version and Date:	01 (7.6.17)

CONFIDENTIAL

The information contained in this document is confidential and the property of the sponsor. The information may not - in full or in part - be transmitted, reproduced, published, or disclosed to others than the applicable Competent Ethics Committee(s) and Regulatory Authority(ies) without prior written authorisation from the sponsor except to the extent necessary to obtain informed consent from those who will participate in the study.

.

Signature Page(s)

Study Title	Optimized **P**erioperative **A**ntibiotic **P**rophylaxis in **Ra**dical **C**ystectomy (PAPRAC)

The Sponsor-Investigator and trial statistician have approved the protocol version [1.1 (dated 29.09.2017)], and confirm hereby to conduct the study according to the protocol, current version of the World Medical Association Declaration of Helsinki, ICH-GCP guidelines or ISO 14155 norm if applicable and the local legally applicable requirements.

Sponsor-Investigator:
Dr.med. Maria Christine Thurnheer Zürcher
Department of Infectious Diseases
University hospital Bern

Bern 30.9.17

Place/Date

Signature

Signature Page(s)

Study Title Optimized **P**erioperative **A**ntibiotic **P**rophylaxis in **Ra**dical **C**ystectomy (PAPRAC)

The Sponsor-Investigator and trial statistician have approved the protocol version [1.1 (dated 29.09.2017)], and confirm hereby to conduct the study according to the protocol, current version of the World Medical Association Declaration of Helsinki, ICH-GCP guidelines or ISO 14155 norm if applicable and the local legally applicable requirements.

Sponsor-Investigator:

Dr.med. Maria Christine Thurnheer Zürcher

Department of Infectious Diseases

University hospital Bern

Place/Date Signature

Trial Statistician:

Andrew Atkinson

Department of Infectious Diseases

University hospital Bern

Bern, 29.09.2017

Place/Date Signature

Local Principal Investigator at study site*:

I have read and understood this trial protocol and agree to conduct the trial as set out in this study protocol, the current version of the World Medical Association Declaration of Helsinki, ICH-GCP guidelines or ISO 14155 norm and the local legally applicable requirements.

Site Bern	Department of Infectious Diseases<br>University Hospital Bern<br>3010 Bern
Principal investigator	PD Jonas Marshall

Bern 29.09.2017

Place/Date — Signature

Site Bern	Department of Urology<br>University Hospital Bern<br>3010 Bern
Principal investigator	Prof. F. Burkhard

Bern 29.09.2017

Place/Date — Signature

Site Bern	Department of Anesthesiology<br>University Hospital Bern<br>3010 Bern
Principal investigator	PD P. Wüthrich

Bern 29.09.2017

Place/Date — Signature

**Note:* In multicentre studies, this page must be individually signed by all participating Local Principal Investigators.

Table of Contents

STUDY SYNOPSIS **9**
STUDY SUMMARY IN LOCAL LANGUAGE CAN BE PROVIDED HERE (GERMAN, FRENCH OR ITALIAN) **16**
ABBREVIATIONS **17**
STUDY SCHEDULE **18**
1. STUDY ADMINISTRATIVE STRUCTURE **20**
1.1 Sponsor, Sponsor-Investigator 20
1.2 Principal Investigator(s) 20
1.3 Statistician ("Biostatistician") 20
1.4 Laboratory 20
1.5 Monitoring institution 21
1.6 Data Safety Monitoring Committee 21
1.7 Any other relevant Committee, Person, Organisation, Institution 21
2. ETHICAL AND REGULATORY ASPECTS **21**
2.1 Study registration 21
2.2 Categorisation of study 21
2.3 Competent Ethics Committee (CEC) 22
2.4 Competent Authorities (CA) 22
2.5 Ethical Conduct of the Study 22
2.6 Declaration of interest 22
2.7 Patient Information and Informed Consent 22
2.8 Participant privacy and confidentiality 22
2.9 Early termination of the study 23
2.10 Protocol amendments 23
3. BACKGROUND AND RATIONALE **24**
3.1 Background and Rationale 24
3.2 Investigational Product (treatment, device) and Indication 25
3.3 Preclinical Evidence 26
3.4 Clinical Evidence to Date 26
3.5 Dose Rationale / Medical Device: Rationale for the intended purpose in study (pre-market MD) 26
3.6 Explanation for choice of comparator (or placebo) 26
3.7 Risks / Benefits 26
3.8 Justification of choice of study population 27
4. STUDY OBJECTIVES **28**
4.1 Overall Objective 28
4.2 Primary Objective 28
4.3 Secondary Objectives 28
4.4 Safety Objectives 28
5. STUDY OUTCOMES **29**
5.1 Primary Outcome 29
5.2 Secondary Outcomes 30
5.3 Other Outcomes of Interest 31
5.4 Safety Outcomes 31
6. STUDY DESIGN **32**
6.1 General study design and justification of design 32

6.2 Methods of minimising bias 33
6.2.1 Randomisation 33
6.2.2 Blinding procedures 33
6.2.3 Other methods of minimising bias..33
6.3 Unblinding Procedures (Code break)..33
7. STUDY POPULATION 34
7.1 Eligibility criteria 34
7.2 Recruitment and screening 34
7.3 Assignment to study groups..34
7.4 Criteria for withdrawal / discontinuation of participants..34
8. STUDY INTERVENTION 36
8.1 Identity of Investigational Products (treatment / medical device)..36
8.1.1 Experimental Intervention (treatment / medical device)..36
8.1.2 Control Intervention (standard/routine/comparator treatment / medical device) 36
8.1.3 Packaging, Labelling and Supply (re-supply) 37
8.1.4 Storage Conditions..37
8.2 Administration of experimental and control interventions 37
8.2.1 Experimental Intervention 37
8.2.2 Control Intervention..37
8.3 Dose / Device modifications..38
8.4 Compliance with study intervention 38
8.5 Data Collection and Follow-up for withdrawn participants 38
8.6 Trial specific preventive measures..38
8.7 Concomitant Interventions (treatments)..38
8.8 Study Drug / Medical Device Accountability 38
8.9 Return or Destruction of Study Drug / Medical Device 39
9. STUDY ASSESSMENTS.. 40
9.1 Study flow chart(s) / table of study procedures and assessments 40
9.2 Assessments of outcomes 42
9.2.1 Assessment of primary outcome..42
9.2.2 Assessment of secondary outcomes 42
9.2.3 Assessment of other outcomes of interest..42
9.2.4 Assessment of safety outcomes 42
9.2.5 Assessments in participants who prematurely stop the study 43
9.3 Procedures at each visit..43
9.3.1 Split into subtitles by type of visit .... **Fehler! Textmarke nicht definiert.**
9.3.2 Split into subtitles by type of visit .... **Fehler! Textmarke nicht definiert.**
9.3.3 Split into subtitles by type of visit .... **Fehler! Textmarke nicht definiert.**
10. SAFETY 46
10.1 Drug studies 46
10.1.1 Definition and assessment of (serious) adverse events and other safety related events ...46
10.1.2 Reporting of serious adverse events (SAE) and other safety related events......................47
10.1.3 Follow up of (Serious) Adverse Events..48
10.2 Medical Device Category C studies .... **Fehler! Textmarke nicht definiert.**
10.2.1 Definition and Assessment of (Serious) Adverse Events and other safety related events**Fehler! Textmarke**
10.2.2 Reporting of (Serious) Adverse Events and other safety related events**Fehler! Textmarke nicht definiert.**

10.2.3 Follow up of (Serious) Adverse Events .... **Fehler! Textmarke nicht definiert.**
10.3 Medical Device Category A studies .... **Fehler! Textmarke nicht definiert.**
10.3.1 Definition and Assessment of safety related events ...... **Fehler! Textmarke nicht definiert.**
10.3.2 Reporting of Safety related events................................ **Fehler! Textmarke nicht definiert.**
11. STATISTICAL METHODS 49
11.1 Hypothesis 49
11.2 Determination of Sample Size 49
11.3 Statistical criteria of termination of trial 49
11.4 Planned Analyses 49
11.4.1 Datasets to be analysed, analysis populations 49
11.4.2 Primary Analysis 50
11.4.3 Secondary Analyses 50
11.4.4 Interim analyses 50
11.4.5 Safety analysis 50
11.4.6 Deviation(s) from the original statistical plan 50
11.5 Handling of missing data and drop-outs 50
12. QUALITY ASSURANCE AND CONTROL 51
12.1 Data handling and record keeping / archiving 51
12.1.1 Case Report Forms 51
12.1.2 Specification of source documents 51
12.1.3 Record keeping / archiving 52
12.2 Data management 52
12.2.1 Data Management System 52
12.2.2 Data security, access and back-up 52
12.2.3 Analysis and archiving 52
12.2.4 Electronic and central data validation 52
12.3 Monitoring 52
12.4 Audits and Inspections 52
12.5 Confidentiality, Data Protection 53
12.6 Storage of biological material and related health data 53
13. PUBLICATION AND DISSEMINATION POLICY 54
14. FUNDING AND SUPPORT 55
14.1 Funding 55
14.2 Other Support 55
15. INSURANCE 55
16. REFERENCES 56
17. APPENDICES 59

STUDY SYNOPSIS

Provide a structured synopsis containing all important information, preferably in tabular view:

**Sponsor / Sponsor-Investigator**	Dr.med. M.C. Thurnheer
**Study Title:**	Optimized Perioperative Antibiotic Prophylaxis in Radical Cystectomy
**Short Title / Study ID:**	PAPRAC
**Protocol Version and Date:**	Version 1, 21.06.2017
**Trial registration:**	This study will be registered at ClinicalTrials.gov and on SNCTP (Swiss National Clinical Trial Portal).
**Study category and Rationale**	Category A
**Clinical Phase:**	Phase 4 study

**Background and Rationale:**	Cystectomy with urinary diversion is the best available treatment option for muscle-invasive bladder cancer. It is also one of the most challenging urological procedures with a high rate of complications. Infections, including surgical site infections, comprise approximately 20% of in-hospital complications. The optimal duration of perioperative antibiotic prophylaxis (PAP) for this type of surgery is unknown. This study aims to clarify the optimal duration of perioperative antibiotic prophylaxis (PAP) in a specific surgical setting. Although guideline recommendations do exist, they are based on low grade evidence, expert opinions, and extrapolations from other fields of abdominal surgery(1, 2). To date no RCTs assessing the optimal duration of perioperative prophylaxis in radical cystectomy have been conducted, the only study addressing the issue was a small non randomized trial by Hara showing that the incidence of SSI was not higher if PAP was given on the day of surgery alone compared to a prolonged prophylaxis.<br><br>Despite the lack of evidence (acknowledged in both publications) 24h PAP is currently recommended in both, European and American society statements. (2) (1) .<br><br>In clinical practice, however, extended use of PAP for more than 48 hours is common: according to current practice at the Urology Department at the Berne University Hospital prophylactic antibiotics are administered for at least 48h post-surgery and usually longer (until all catheter/stents are removed).<br><br>Inconsistent adherence to PAP recommendations and guidelines is not a problem specific to Bern but is observed internationally in both American and European settings (3) (4). This behaviour is very likely linked to the lack of strong evidence in the setting of a challenging surgical procedure (5) (4).<br><br>We propose a single center, randomized, controlled clinical non-inferiority trial to assess the efficacy and safety of the recommended 24h PAP in radical cystectomy, compared to the currently prescribed extended prophylaxis. As primary endpoints, both the rate of surgical site infections (SSI) and the rate of urinary tract infections will be assessed. In addition, antibiotic-associated adverse events, the rate of C. difficile colitis directly antibiotic-related costs and crude mortality, will be evaluated for each trial arm.<br><br>Evaluating the viability of a 24h PAP for radical cystectomy with urinary deviation has important practical implications:<br><br>Our findings will provide currently lacking evidence for the risks and benefits of short-term vs. extended PAP, allowing for a rational approach to the use of prophylactic antibiotics in a frequently performed urological procedure.<br><br>Our findings will either allow to reduce or to justify relevant monetary costs and health-associated expenditures (side effects, development of microbial resistance).<br><br>In light of the Federal Office of Public Health's recently enacted strategy to combat antimicrobial resistance (StAR), safely reducing antimicrobial consumption for prophylaxis could be a significant contribution to these national efforts.

**Objective(s):**	Research hypothesis<br>Short-term perioperative antibiotic prophylaxis (24h) is non-inferior to the currently used extended perioperative prophylaxis in preventing surgical site infections.<br>**Research Objectives**<br>1. To investigate whether the recommended short-term perioperative PAP is non-inferior to the currently used extended PAP in patients undergoing radical cystectomy with urinary diversion.<br>2. To evaluate if there are clinical sequelae associated with extended vs. 24h PAP.<br>3. To examine the effects of extended vs. 24h PAP on the local microflora and on the selection of resistant bacteria.<br>4. To assess the monetary costs of extended vs. 24h PAP
**Outcome(s):**	**Primary endpoints:**<br>• Rate of surgical site infections and rate of urinary tract infections in patients receiving short-term vs. extended antibiotic prophylaxis (objective 1).<br>**Secondary endpoints:**<br>• Rates of antibiotic-associated adverse events, rate of C. difficile colitis and crude mortality in patients receiving short-term vs. extended antibiotic prophylaxis (objective 2).<br>• Frequency of (multi-) resistant bacteria in subsequent urine samples (objective 3).<br>• Changes in the fecal microflora after short-term vs. extended PAP (sub-study, objective 3)<br>Directly antibiotic-associated costs incurred during short-term vs. extended antibiotic prophylaxis (objective 4).
**Study design:**	The efficacy and safety of 24h PAP (≤24h) vs. the current practice of extended PAP (Prophylactic antibi>48h, until all catheter/stents are removed) will be compared in a randomized controlled clinical non-inferiority trial.

**Inclusion / Exclusion criteria:**	Patients (or a representative) must provide written, informed consent before any study procedures occur<br>Inclusion criteria:<br>Age >18 years<br>Planned radical cystectomy at the Department of Urology, Bern University Hospital<br>Exclusion criteria:<br>• Contraindications to the classes of drugs under study, e.g. known hypersensitivity or allergy to class of drugs including alternatives described in the protocol or the investigational product,<br>• Women who are pregnant or breast feeding (exclusion for surgery),<br>• Inability to follow the procedures of the study, e.g. due to language problems, psychological disorders, dementia, etc. of the participant,<br>• Previous enrolment into the current study,<br>• Enrolment of the investigator, his/her family members, employees and other dependent persons,<br>As the current protocol does not involve any investigational drugs or procedures, patients are eligible for dual randomization if they are on stable doses of the investigational drug and there are no expected interactions with the antibiotics used for perioperative prophylaxis

<table>
<tr><td>Measurements and procedures:</td><td>
Allocation of eligible patients will be performed by concealed block randomization (computer based), stratified for age and gender, and equal proportions will be assigned to the control (extended PAP) and intervention (24h PAP) group.
The parenteral antibiotic regimen will be the same for the two groups and reflects the current clinical practice at the Department for Urology, the only difference being the duration of prophylactic treatment:
<table>
<tr><td>Substance</td><td>Dose/day</td><td colspan="2">Duration</td></tr>
<tr><td></td><td></td><td>24h PAP</td><td>Extended PAP</td></tr>
<tr><td>Tobramycin</td><td>120mg iv single dose pre-surgery</td><td>x</td><td>x</td></tr>
<tr><td>Tobramycin</td><td>80mg iv q8h</td><td>24h</td><td>48h</td></tr>
<tr><td>Metronidazole</td><td>1000mg single dose pre-surgery</td><td>x</td><td>x</td></tr>
<tr><td>Metronidazole</td><td>500mg iv q12h</td><td>24h</td><td>48h</td></tr>
<tr><td>Amoxicillin/Clavulanate</td><td>1.2g/iv q8h</td><td>24h</td><td>Until all catheter/stents are removed</td></tr>
<tr><td colspan="4">
Dosages will be adapted to renal functions and -if indicated- to patients weight; renal and neural toxicities will be monitored
In patients allergic to penicillin or beta lactam antibiotics, amoxicillin/clavulanate will be replaced by a combination of vancomycin (2x1g/24h, or adapted to creatinine clearance) plus ciprofloxacin 500mg
In patients colonized with resistant bacteria prior to surgery, the antibiotic regimen will be adapted according to the documented antibiotic sensitivities.
The first dose of antibiotics will be administered within 1 hour before incision.
Antibiotics will be stopped 24h post-surgery in the 24h PAP-group and after >48h (when all catheter/stents are removed) in the extended PAP group. A study nurse will call the treating team after 24h to ensure adherence to protocol (explicit question to treating physician: “did you stop the perioperative prophylaxis”) for the short-term group.
</td></tr>
</table>
</td></tr>
<tr><td>Study Product / Intervention:</td><td>Perioperative antibiotic prophylaxes of 24h post-surgery as recommended by clinical guidelines, (24h PAP) (1) (1)</td></tr>
<tr><td>Control Intervention (if applicable):</td><td>Extended perioperative antibiotic prophylaxis (>48h post-surgery or until all catheter/stents are removed) according to current clinical practice (extended PAP)</td></tr>
</table>

**Number of Participants with Rationale:**	Sample size calculation<br><br>We define the proportion of surgical site infections (SSI) after 30 days in the control group (>48h PAP) to be pC and the proportion of SSI for shorter period (≤ 24h) pE. The null hypothesis is defined as the 95% confidence for the difference in rates (pC - pE) being within the interval of therapeutic equivalence (D). Formally, H0: (pC - pE) > D and H1: (pC - pE) < D. The margin D was defined based on clinical and statistical reasoning: The local rate of SSIs at the Urology Clinic with currently >48h days of PAP is not known. Based on available literature, we assumed a baseline SSI rate of 10% with standard treatment and an increased risk of 15% SSI in the intervention group, with the difference being approximately 5%. Due to the large variation in reported SSI incidence we define the interval of therapeutic difference to be 2 times the difference found by Hara et al (12), i.e., D = 0.07 (93 patients/group). Taking into account a reported post-randomization dropout rate of 7% (6/83), we dimensioned the sample size for the per-protocol analysis including this inflation factor to be 186/(1-0.07) = 196 patients with 98 patients in each group. Based on a mean frequency of 79 radical cystectomies performed annually (2010-2015) a study duration of three years is projected.

Study Duration:

Research outline/Milestones			
Month	Milestone	Objective	Who
0- 36 (36months)	Patient recruitment, randomization, Data collection	Clinical trial	Clinical team (Wüthrich/Burkha SN
12*	Interim analysis I	Verifying sample size calculation, interim outcome (primary endpoints), safety evaluation	Atkinson /Thurnheer Marschall
24**	Interim analysis II		
36	Inclusion of 98 patients/trial arm	Closing of study	Thurnheer/ SN /Marschall Burk /Wüthrich
42-48	Analysis of data Publication of data	Distribute findings, change of current PAP strategy if supported by results	Thurnheer Atkinson Marschall Burkhard Wüthrich
42-48	Sub-study: changes in microbiome	Assessing changes in the fecal microbiota associated with different PAP strategies	Thurnheer

* after recruitment of 70 patients, i.e., half of projected study population

**after recruitment of 140 patients

SN=study nurse

Study Schedule:

12/17

12/20

**Investigator(s):**	Dr.med. M.C. Thurnheer (Sponsor Investigator)<br>PD Jonas Marschall (Principal Investigator)<br>Prof Fiona Burkhard (Investigator)<br>PD Patrick Wüthrich (Investigator)<br>Andrew Atkinson (Trial statistician)
**Study Centre(s):**	Universitätsklinik für Urologie,<br>Inselspital<br>3010 Bern<br>Switzerland
**Statistical Considerations:**	Descriptive statistics will be produced based on mean and standard deviation for continuous, normally distributed variables; median and inter-quartile range for continuous, non-normally distributed variables; and percentages of the appropriate marginal totals for dichotomous and other categorical variables. The Shapiro-Wilk test will be used to investigate departures from normality for continuous variables.<br>Group differences will be tested using the t-test or analysis of variance (ANOVA) methods for continuous, normally distributed variables, the Wilcoxon-Mann-Whitney or Kruskal-Wallis tests for continuous, non-normally distributed variables, and the chi-square (respectively proportions) test for dichotomous and categorical variables. Wald-type confidence intervals will be used for dichotomous and categorical variables.<br>A significance level of 5% will used throughout the study, Bonferroni corrected for multiple testing where appropriate.
**GCP Statement:**	This study will be conducted in compliance with the protocol, the current version of the Declaration of Helsinki, the ICH-GCP or ISO EN 14155 (as far as applicable) as well as all national legal and regulatory requirements.

STUDY SUMMARY IN LOCAL LANGUAGE

Bei grösseren chirurgischen Eingriffen werden vorbeugend Antibiotika verabreicht. Diese Antibiotikaprophylaxe hilft, Infektionen, die in Zusammenhang mit dem chirurgischen Eingriff stehen zu verhindern. Allerdings ist bei vielen Eingriffen unklar, wie lange diese Prophylaxe eingesetzt werden sollte, es gibt wenig Untersuchungen die sich mit dieser Frage beschäftigen. Oft werden vorbeugende Antibiotika unnötig lange verschrieben. Dies kann zu einer vermehrten Resistenzbildung von Bakterien führen und eine erhöhte Anzahl von Nebenwirkungen beim Patienten zur Folge haben.

Die hier präsentierte Studie PAPRAC soll klären, wie lange die Antibiotikaprophylaxe bei einer bestimmten Gruppe von urologischen Operationen dauern sollte: Gemäss aktuellen Empfehlungen sollte bei der radikalen Zystektomie (komplette chirurgische Blasenentfernung mit Anlegen einer Harnabletung) die Antibiotikaprophylaxe 24 Stunden nach dem Eingriff gestoppt werden. In der klinischen Praxis wird aber eine deutlich längere Dauer der Antibiotikaprophylaxe verschrieben (mindestens 48 Stunden und oft 5-7 Tage). Diese verlängerte Prophylaxedauer kann zu einer vermehrten Resistenzentwicklung der Bakterien und auch zu Komplikationen in Zusammenhang mit den Medikamenten (Unverträglichkeitsreaktionen, Infektionen im Bereich der Einstichstelle bei intravenöser Gabe) führen, der Nutzen einer verlängerten Prophylaxe ist hingegen unklar.

Im Rahmen der Studie werden zwei Prophylaxeformen untersucht: Eine Gruppe von Patienten wird gemäss gängiger Praxis behandelt und eine verlängerte Prophylaxe von mindestens 48 Stunden erhalten. Die andere Gruppe wird eine den offiziellen Empfehlungen entsprechende verkürzte Prophylaxedauer erhalten. Die teilnehmende Patienten nach dem Zufallsprinzip einer der beiden Gruppen zugewiesen.

Als Studienendpunkt wird die Häufigkeit von mit dem chirurgischen Eingriff in Zusammenhang stehenden Infekten (Wundinfektionen und Harnwegsinfektionen) untersucht, die Häufigkeit von Nebenwirkungen und die Kosten in beiden Gruppen werden ebenfalls analysiert.

ABBREVIATIONS

Provide a list of abbreviations used on the protocol - to be completed

AE	Adverse Event
CA	Competent Authority (e.g. Swissmedic)
CEC	Competent Ethics Committee
CRF	Case Report Form
ClinO	Ordinance on Clinical Trials in Human Research *(in German: KlinV, in French: OClin)*
eCRF	Electronic Case Report Form
CTCAE	Common terminology criteria for adverse events
DSUR	Development safety update report
GCP	Good Clinical Practice
IB	Investigator's Brochure
Ho	Null hypothesis
H1	Alternative hypothesis
HFG	Humanforschungsgesetz (Law on human research)
HMG	Heilmittelgesetz
HRA	Federal Act on Research involving Human Beings
IMP	Investigational Medicinal Product
IIT	Investigator-initiated Trial
ISO	International Organisation for Standardisation
ITT	Intention to treat
KlinV	Verordnung über klinische Versuche in der Humanforschung *(in English: ClinO, in French OClin)*
LPTh	Loi sur les produits thérapeutiques
LRH	Loi fédérale relative à la recherche sur l'être humain
MD	Medical Device
OClin	Ordonnance sur les essais cliniques dans le cadre de la recherche sur l'être humain *(in German : KlinV, in English : ClinO)*
PAP	Perioperative antibiotic prophylaxis
PI	Principal Investigator
SDV	Source Data Verification
SOP	Standard Operating Procedure
SPC	Summary of product characteristics
SUSAR	Suspected Unexpected Serious Adverse Reaction
TMF	Trial Master File

STUDY SCHEDULE										
TIMEPOINT	Screening	Baseline (surgery)	T1	T2	T3	T4	T5	T6	T7	T8
Day/h	-10d - -1d	0	1d 24h	2d 48h	3d 72h	5d	7d	10d (+4d)	30d (+7d)	90d (+/-7d)
SCREENING/BASELINE										
Patient information and informed consent	x									
Inclusion/Exclusion criteria	x									
Demographic data[1)]	x									
Medical history [2)]	x									
Colonization with MDR bacteria?	x									
Concommitant medication	x	x								
Physical examination (T, BD, HR, weight, height)	x	x								
Blood examination [3)]	x	x								
Urine examination [4)]										
Microbiology [5)]		x								
Stool sample	x									
Randomization/Allocation		x								
Adverse Events	x	x								
INTERVENTION										
Short term PAP [6)]			***							
Extended PAP [7)]										
Outcome ASSESSMENT										
SSI [8)]					x	x	x	x	x	x
Adverse Events [10)]			x	x	x	x	x	x	x	x
Clinical outcome variables [9)]			x	x	x	x	x	x	x	
Concomitant medication			x	x	x	x	x	x	x	
Physical examination (T, BD, HR, weight, height)			x	x	x	x	x	x	x	
Blood examination [3)]			x	x	x	x	x	x	x	
Urine examination [4)]			x	x	x	x	x	x	x	x
Microbiology [5)]	x				x		x	x	x	x
Stool sample	x							x	x	
Total days of antibiotics prescribed									x	x
Total days of prophylactic ABx									x	x
Costs of antibiotics									x	x
Number of inpatient days									x	x

*at pre-op assessment
***Tel study nurse (explicit question to treating physician: “did you stop the perioperative prophylaxis”)

1) age, gender, ethnicity, language
2) current Diagnosis, Diabetes, smoking, heart disease, hypertension, previous UTI
3) Hematogram V, CRP, Creatinine, Urea, ALAT ASAT
4) Urine chemistry
5) Results of urine culture
6) 24h prophylaxis
7)>48h prophylaxis (stop when all catheter/stents are removed)
8) signs of SSI (if yes further classified)
9) According to CRF: Vital signs (T, BP, HR) , signs of blood stream infection (yes/no), signs of UTI (yes/no),
10) signs of antibiotic-related AE (nausea, diarrhea, allergic reaction (skin rash or other), hepatotoxicity (ASAT, ALAT >2xULN), renal toxicity (Creatinine >150umol/L or >50% increase from baseline)) line associated infections? Diarrhea yes/no (if yes: Detection of C.difficile?)

1. STUDY ADMINISTRATIVE STRUCTURE

1.1 Sponsor, Sponsor-Investigator

Dr. med. Maria ChristineThurnheer

Department of Infectious Diseases

University Hospital Bern

3010 Bern, Switzerland

Email: Christine.Thurnheer@insel.ch

Tel: ++4131 632 21 11

Role: Main author of study protocol, responsible for study design, data analysis, study report, preparation of manuscript

1.2 Principal Investigator(s)

PD Dr. med. Jonas Marschall

Department of Infectious Diseases

University Hospital Bern

3010 Bern, Switzerland

email Jonas.Marschall@insel.ch

Tel: ++41 31 632 21 11

Role: Co-author of study protocol, senior investigator at study site, co-author of data analysis, co-author of study report

Prof. Dr. med. Fiona Burkhard

Department of Urology

University Hospital Bern

3010 Bern, Switzerland

Role: co-authors of study protocol, co-investigators, co-authors of study report and manuscript

PD Dr. med. Patrick Wüthrich

Department of Anesthesiology

University Hospital Bern

3010 Bern, Switzerland

Role: co-investigator, co-authors of study report and manuscript

1.3 Statistician ("Biostatistician")

Andrew Atkinson, MA

Department of Infectious Diseases

University Hospital Bern

3010 Bern, Switzerland

Tel: 41 31 63 2 69 68

Email: Andrew.Atkinson@insel

1.4 Laboratory

Zentrum für Labormedizin, Bern University Hospital, 3010 Bern, Switzerland

Institut für Infektionskrankheiten, University of Bern, Freiburgstrasse, 3010 Bern, Switzerland

1.5 Monitoring institution

Department of Infectious Diseases
Monitoring study nurse, Melanie Lacalamita
University Hospital Bern
Bern, Switzerland

1.6 Data Safety Monitoring Committee

Not applicable

1.7 Any other relevant Committee, Person, Organisation, Institution

Not applicable

2. ETHICAL AND REGULATORY ASPECTS

Before the study will be conducted, the protocol, the proposed patient information and consent form as well as other study-specific documents shall be submitted to a properly constituted Competent Ethics Committee (CEC) in agreement with local legal requirements, for formal approval. Any amendment to the protocol must as well be approved (if legally required) by these institutions.

The decision of the CEC concerning the conduct of the study will be made in writing to the Sponsor-Investigator before commencement of this study. The clinical study can only begin once approval from all required authorities has been received. Any additional requirements imposed by the authorities shall be implemented.

2.1 Study registration

This study will be registered at ClinicalTrials.gov and on SNCTP (Swiss National Clinical Trial Portal).

2.2 Categorisation of study

This study is categorized "other clinical trial" , risk category A:
Chapter 4 Other Clinical Trials

KLinV Section 1 General Provisions
Art. 60 Scope
This Chapter applies to clinical trials which are neither trials of therapeutic products or transplant products nor trials of transplantation.
Art. 61 Categorisation
1 A clinical trial comes under Category A if the health-related intervention investigated:
a. entails only minimal risks and burdens; or
b. is recognised as standard in guidelines prepared in accordance with internationally accepted quality criteria.
2 A clinical trial comes under Category B if the health-related intervention investigated:
a. entails more than minimal risks and burdens; and
b. is not recognised as standard as specified in paragraph 1 letter b.

2.3 Competent Ethics Committee (CEC)

Approval for this study is sought at the KEK Bern and reporting duties and time frame will be performed as requested by KEK Bern. No changes are made to the protocol without prior Sponsor and CEC approval, except where necessary to eliminate apparent immediate hazards to study participants.

Premature study end or interruption of the study is reported within 15 days. The regular end of the study is reported to the CEC within 90 days, the final study report shall be submitted within one year after study end. Amendments are reported according to chapter 2.10.

2.4 Competent Authorities (CA)

Risk category A-> no Swissmedics approval needed

2.5 Ethical Conduct of the Study

The study will be carried out in accordance to the protocol and with principles enunciated in the current version of the Declaration of Helsinki, the guidelines of Good Clinical Practice (GCP) issued by ICH, in case of medical device: the European Directive on medical devices 93/42/EEC and the ISO Norm 14155 and ISO 14971, the Swiss Law and Swiss regulatory authority's requirements. The CEC and regulatory authorities will receive annual safety and interim reports and be informed about study stop/end in agreement with local requirements.

2.6 Declaration of interest

The study investigators have no intellectual, financial, proprietary conflict of interest to declare.

2.7 Patient Information and Informed Consent

The investigators will explain to each participant the nature of the study, its purpose, the procedures involved, the expected duration, the potential risks and benefits and any discomfort it may entail. Each participant will be informed that the participation in the study is voluntary and that he/she may withdraw from the study at any time and that withdrawal of consent will not affect his/her subsequent medical assistance and treatment.

The participant must be informed that his/her medical records may be examined by authorised individuals other than their treating physician.

All participants for the study will be provided a participant information sheet and a consent form describing the study and providing sufficient information for participant to make an informed decision about their participation in the study. Participants will have acceptable ample time and opportunity to inquire about details of the trial and to decide whether or not to participate in the trial.

The patient information sheet and the consent form will be submitted to the CEC to be reviewed and approved. The formal consent of a participant, using the approved consent form, must be obtained before the participant is submitted to any study procedure.

The participant should read and consider the statement before signing and dating the informed consent form, and should be given a copy of the signed document. The consent form must also be signed and dated by the investigator (or his designee) and it will be retained as part of the study records.

2.8 Participant privacy and confidentiality

The investigator affirms and upholds the principle of the participant's right to privacy and that they shall comply with applicable privacy laws. Especially, anonymity of the participants shall be guaranteed when presenting the data at scientific meetings or publishing them in scientific journals.

Individual subject medical information obtained as a result of this study is considered confidential and disclosure to third parties is prohibited. Subject confidentiality will be further ensured by utilising subject identification code numbers to correspond to treatment data in the computer files.

For data verification purposes, authorised representatives of the Sponsor (-Investigator), a competent authority (e.g. Swissmedic), or an ethics committee may require direct access to parts of the medical records relevant to the study, including participants' medical history.

2.9 Early termination of the study

The Sponsor-Investigator may terminate the study prematurely according to certain circumstances, such as:

- ethical concerns,
- insufficient participant recruitment,
- when the safety of the participants is doubtful or at risk, respectively,
- alterations in accepted clinical practice that make the continuation of a clinical trial unwise,
- early evidence of benefit or harm of the experimental intervention

2.10 Protocol amendments

Substantial amendments are only implemented after approval of the CEC and CA respectively.

Under emergency circumstances, deviations from the protocol to protect the rights, safety and well-being of human subjects may proceed without prior approval of the sponsor and the CEC/CA. Such deviations shall be documented and reported to the sponsor and the CEC/CA as soon as possible.

All Non-substantial amendments are communicated to the CA as soon as possible if applicable and to the CEC within the Annual Safety Report (ASR).

3. BACKGROUND AND RATIONALE .

3.1 Background and Rationale

Cystectomy with urinary diversion (ileal conduit, orthotopic ileal bladder substitute, continent catheterizable pouch) is the best treatment option for patients with muscle-invasive bladder cancer. This intervention is one of the most challenging in urology and has a high rate of postoperative complications of 50-60%, including up to 30% of postoperative infections (6, 7).

Perioperative antibiotic prophylaxis (PAP) is widely accepted as a crucial preventive measure to reduce the incidence of surgical site infections (SSI) (8, 9). The rationale for PAP is the reduction of the local bacterial load at the site and time of intervention, and therefore a short duration of PAP of 24 to maximal 48 hours is recommended for all clean to clean-contaminated procedures (2, 3). In situations where either a local infection has been present prior to surgery, or when compartments with high bacterial load have to be entered (contaminated to dirty procedures), antibiotics are continued as preemptive treatment (not prophylaxis) after surgery.

Although guideline recommendations do exist, they are based on low grade evidence, expert opinions, and extrapolations from other fields of abdominal surgery(1, 2). To date no RCTs assessing the optimal duration of perioperative prophylaxis in radical cystectomy have been conducted, and therefore high level evidence supporting supporting the optimal duration of PAP in radical cystectomy with urinary diversion is lacking (10-12).The only study addressing the issue was a small non randomized trial by Hara showing that the incidence of SSI was not higher if PAP was given on the day of surgery alone compared to a prolonged prophylaxis.

Evidence Based on data extrapolated from abdominal surgery, current guidelines recommend 24h PAP (≤24h) for all clean-contaminated procedures including radical cystectomy (1, 10, 13, 14) and this is also recommended in both, European and American Urology society statements. (2) (1) .

Adherence to PAP recommendations and guidelines in general is inconsistent and often very limited. A recent evaluation revealed a significant inter-hospital variability of PAP and showed that extended use (>48h) was common in patients undergoing radical cystectomy (15). Importantly, this study also demonstrated that longer duration of PAP incurred higher costs and was associated with an increased rate of C. difficile colitis. A small, prospective, non-randomized study showed equal efficacy of 24h PAP in preventing postoperative infections in patients undergoing radical cystectomy with ileum conduit compared to extended PAP (16). Nonetheless, larger randomized clinical trials supporting these findings are lacking. Not surprisingly retrospective cohort study, published in 2017 showed a high heterogeneity of antibiotic prophylaxis practices in the context of radical cystectomy and a poor adherence to published guidelines(4) . This is not a problem specific to Bern but is observed internationally in both American and European settings (3) (4). This behavior is very likely linked to the lack of strong evidence in the setting of a challenging surgical procedure (5) (4).

With the evidence generated by the proposed study a 24h PAP approach in radical cystectomy will either be substantiated or can be discarded. Using an evidence based, rational approach to antibiotic prescription is important on both a societal and individual level due to the following aspects:

The unwarranted extended use of antibiotics is a major concern as exposure to antibiotics is a driving force for the development of (multi-) resistant bacteria and will lead to an increasing number of difficult-to-treat infections with challenging clinical, financial and ecological consequences (17) (18).

Resistance development of microorganisms is closely linked to misguided use of antibiotics and emerging multi-resistant bacteria are a direct outcome of a lack of antibiotic stewardship (17, 19).

Thishas been recognized on both national and international levels and is addressed within antimicrobial stewardship frameworksOn a hospital level prophylactic use of antibiotics is an important contributor to the overall antibiotic consumption. A recent point prevalence study at the Inselspital Bern demonstrated 20% of all antibiotics prescribed were used for surgical prophylaxis and 15 % of PAP was prescribed for longer than 24h (personal communication Dr.R. Sommerstein, Oberarzt Spitalhygiene Inselspital Bern). Optimizing current practices of PAP will have a direct impact on overall antibiotic consumption in a specific hospital.

Cystectomy is distinct from other types of surgery as there are multiple catheters remaining in situ for a prolonged period, often more than 10 days (ureteral stents, suprapubic and transurethral catheter).

Bacterial colonization of these stents is to be expected and occurs regularly and antibiotics are often applied until removal of all catheters.

Approximately 70-80 radical cystectomies with urinary diversion are performed annually at the Department of Urology at Bern University Hospital. Prophylactic antibiotics are routinely used for >48h and are continued until all catheter/stents are removed, resulting in an average duration of 3-9 days (personal communication Prof. Burkhard) as opposed to the 24h of prophylaxis formally recommended by European and US guidelines. This results in a possibly unnecessary overuse of antibiotics with an associated risk of drug-related adverse events, extended selection pressure on the local microflora with the potential for resistance development, and the generation of additional direct and indirect costs

The proposed RCT will determine if the recommended 24h prophylaxis is non-inferior to the current approach used in Bern and by otherelsewhere s to prevent surgical site infections and urinary tract infections in these patients.
Patients will be closely monitored and thus are not likely to be at a disadvantage. On the other hand future patients may benefit from the knowledge gained from a well-designed study. There are no invasive interventions and patients are not exposed to any additional disagreeable examinations.

.

We hope the proposed study will either allow reducing inappropriate prescription (less exposure to antibiotics, less AEs, lower pressure on the local and hospital microflora with individual and institutional benefits) and lower monetary costs. (15)), or it will justify a prolonged PAP course in a specific situation based on good quality evidence.

.

3.2 Investigational Product (treatment, device) and Indication

This trial will examine whether the duration of perioperative antibiotic prophylaxis (PAP) can be safely shortened to 24h postsurgery as recommended by current guidelines. All antibiotics used in this study are approved and will be used according to the current labelling by the competent authorities (Swissmedics):

Substance	Dose/day	Duration	
		24h PAP	Extended PAP
Tobramycin	120mg iv single dose pre-surgery	x	x
Tobramycin	80mg iv q8h	24h	48h
Metronidazole	1000mg single dose pre-surgery	x	x
Metronidazole	500mg iv q12h	24h	48h
Amoxicillin/Clavulanate	1.2g/iv q8h	24h	Until all catheter/stents are removed
Dosages will be adapted to renal functions and -if indicated- to patients weight; renal and neural toxicities will be monitored<br>In patients allergic to penicillin or beta lactam antibiotics, amoxicillin/clavulanate will be replaced by a combination of vancomycin (2x1g/24h, or adapted to creatinine clearance) plus ciprofloxacin 500mg<br>In patients colonized with resistant bacteria prior to surgery, the antibiotic regimen will be adapted according to the documented antibiotic sensitivities.<br>The first dose of antibiotics will be administered within 1 hour before incision.<br>Antibiotics will be stopped 24h post-surgery in the 24h PAP-group and after >48h (when all catheter/stents are removed) in the extended PAP group. A study nurse will call the treating team after 24h to ensure adherence to protocol (explicit question to treating physician: "did you stop the perioperative			

prophylaxis") for the short-term group.

3.3 Preclinical Evidence

Not applicable

3.4 Clinical Evidence to Date

Perioperative antibiotic prophylaxis (PAP) is widely accepted as a crucial preventive measure to reduce the incidence of surgical site infections (SSI) (8, 9). The rationale for PAP is the reduction of the local bacterial load at the site and time of intervention, and therefore a short duration of PAP of 24 to maximal 48 hours is recommended for all clean to clean-contaminated procedures. In situations where either a local infection has been present prior to surgery, or when compartments with high bacterial load have to be entered (contaminated to dirty procedures), antibiotics are continued as preemptive treatment (not prophylaxis) after surgery.

Evidence supporting the optimal duration of PAP for radical cystectomy with urinary diversion is lacking (10-12). Based on data extrapolated from abdominal surgery, current guidelines recommend 24h PAP (≤24h) for all clean-contaminated procedures including radical cystectomy (1, 10, 13, 14).

However, a recent evaluation revealed a significant inter-hospital variability of PAP and showed that extended use (>48h) was common in patients undergoing radical cystectomy (15). Importantly, this study also demonstrated that longer duration of PAP incurred higher costs and was associated with an increased rate of C. difficile colitis. A small, prospective, non-randomized study showed equal efficacy of 24h PAP in preventing postoperative infections in patients undergoing radical cystectomy with ileum conduit compared to extended PAP (16). Nonetheless, larger randomized clinical trials supporting these findings are lacking.

3.5 Dose Rationale / Medical Device: Rationale for the intended purpose in study (pre-market MD)

Route of administration and dosage are in accordance with drug registration and labelling by the competent authorities (Swissmedics) and reflect either current clinical practice (extended PAP arm) or current guideline recommendations (short PAP arm)

3.6 Explanation for choice of comparator (or placebo)

The aim of this study is to compare the current clinical praxis (extended PAP) with a shortened duration (24h PAP) of perioperative antibiotic prophylaxis in patients undergoing radical cystectomy. The rational for assessing the safety of a 24h perioperative antibiotic prophylaxis compared to the currently used extended prophylaxis is based on current guideline recommendations. Further reduction of the duration might put patients at additional risks, further prolongation of prophylaxis would not be in accordance with current recommendations and strategies regarding antibiotic stewardship.

3.7 Risks / Benefits

General risks/benefits: Perioparative antibiotic prophylaxis is widely recognized as integral part of a bundled prevention strategy to reduce the incidence of surgical site infections. Administration of intravenous antibiotics can cause directly drug-related adverse events (drug toxicity (hepatotoxicity, renal toxicity, ototoxicity, hematotoxicity, gastrointestional adverse events (nausea, diarrhea, C.difficile colitis)), allergic reactions (immediate reaction delayed reaction), and adverse events associated with the route of administration (pain/inflammation at the site of line insertion, inadvertent perivascular drug administration, line associated thrombophlebitis, line associated infection)

24h PAP: Although supported by current surgical guidelines, randomised controlled clinical trials

investigating the safety of shortened PAP after radical cystectomy are lacking. Therefore shortened duration of PAP may lead to a higher frequency of surgical site infections. This aspect will be assessed as primary outcome of the study and will be closely monitored.

Extended PAP: The unwarranted extended use of antibiotics can lead to the development of (multi-) resistant bacteria and to an increasing number of difficult-to-treat infections (13) (14). Also the risk of developing unwarranted side effects directly or indirectly related to the intravenous administration of antibiotics may be increased by extended exposure to these substances.

Evaluating the viability of a 24h PAP for radical cystectomy with urinary deviation has important practical implications:

Our findings will provide currently lacking evidence for the risks and benefits of short-term vs. extended PAP, allowing for a rational approach to the use of prophylactic antibiotics in a frequently performed urological procedure.

Our findings will either allow to reduce or to justify relevant monetary and health-associated (side effects, development of microbial resistance) costs.

In light of the Federal Office of Public Health's recently launched strategy to combat antimicrobial resistance (StAR), safely reducing antimicrobial consumption for prophylaxis could be a significant contribution to these national efforts.

3.8 Justification of choice of study population

The aim of this study is to investigate whether reducing the duration of perioperative prophylaxis in radical cystectomy is effective and safe. Therefore all patients, >18years old ., undergoing radical cystectomy at the Department of Urology at the Bern University hospital are eligible for this study.

4. STUDY OBJECTIVES

4.1 Overall Objective

Research hypothesis

Short-term perioperative antibiotic prophylaxis (24h) is non-inferior to the currently used extended perioperative prophylaxis in preventing surgical site infections.

4.2 Primary Objective

This study seeks to determine whether short-term perioperative antibiotic prophylaxis (24h) is non-inferior to the currently used extended perioperative prophylaxis in preventing surgical site infections in patients undergoing radical cystectomy.

Primary objective:

- To assess the both the rate of surgical site infections and rate of urinary tract infections* in patients receiving short term vs. extended antibiotic prophylaxis.

*During the surgical procedures for radical cystectomy with urinary diversion both the gastrointestinal tract and the urinary tract are accessed. Therefore we will evaluate rates of urinary tract infections in addition to classical surgical site infections (i.e. wound- or incision- associated infections)

Secondary objectives:

- To evaluate rates of antibiotic-associated adverse events, rate of C. difficile colitis and crude mortality in patients receiving short-term vs. extended antibiotic prophylaxis.
- To examine the rates of (multi-) resistant bacteria in subsequent urine samples at predefined time points.
- To assess directly antibiotic-associated costs incurred during short-term vs. extended antibiotic prophylaxis
- To examine changes in the fecal microflora after short-term vs. extended PAP (sub-study

4.3 Safety Objectives

The primary study endpoint (rate/incidence of SSI) is the main safety objective. In addition the following safety objectives will be assessed in the two study arms:

- rates of antibiotic-associated adverse events (direct organotoxicity, gastrointestinal side effects, hypersensitivity reactions, indirect AEs such as line-associated inflammation/ infection)
- rate of C. difficile colitis
- crude mortality.
- Frequency of (multi-) resistant bacteria in subsequent urine samples.

5. STUDY OUTCOMES

5.1 Primary Outcome

Primary endpoints:

- Rate of surgical site infections and rate of urinary tract infections in patients receiving short term vs. extended antibiotic prophylaxis

Assessment: Occurrence of surgical site infections according to current definitions (see below) will be assessed at day 10 and 30 post surgery by clinical and laboratory evaluation performed by the treating physician. In addition to the event rate, the time point off SSI diagnosis will be recorded to allow for later time to event analysis.

Surgical site infections (SSI) will be diagnosed as defined by the Centers for Disease Control and Prevention criteria (20) (CDC Procedure associated Module, Surgical Site Infection Event, January 2017).

1) **Superficial surgical site infection:**
Date of event within 30and 90 days of the operative procedure (where day 1= day of procedure), involves only skin and subcutaneous tissue of the incisions AND patient has at least one of the following:

a) purulent drainage from the superficial incision.

b) Organisms identified from an aseptically obtained specimen from the superficial incision or subcutaneous tissue by a culture or non-culture based microbiological testing method which is performed for clinical diagnosis or treatment.

c) Superficial incision is deliberately opened by a surgeon, attending physician or other designee and microbiological texting is not performed AND patient has at least one of the following signs and symptoms: pain or tenderness, localized swelling, erythema or heat.

d) SSi is clinically diagnosed by a surgeon, attending physician or other designee

2) **Deep incisional Surgical site infections:**

Date of event within 30 and 90 days of the operative procedure (where day 1= day of procedure), involves deep soft tissue of the incision (fascial and muscle layers) AND patient has at least one of the following:

a) purulent drainage from the deep incision.

b) a deep incision that spontaneously dehisces or is deliberately opened by a surgeon, attending physician or other designee and organism is identified by a culture or non-culture based microbiological testing method which is performed for clinical diagnosis or treatment

c) a deep incision that spontaneously dehisces or is deliberately opened by a surgeon, attending physician or other designee no microbiological disgnostic is performed AND patient hast at least one of the following: Temperature >38°C, localized pain or tenderness (if microbiological diagnostic test is performed and shows a negative finding this criteria is not fulfilled)

d) an abscess or other evidence of infection involving deep incision is detected on gross anatomical or histopatological exam or imaging test.

3) **Organ/Space Surgical site infection:** Date of event within 30 and 90 days of the operative procedure (where day 1= day of procedure), involves any part of the body deeper than the fascial/muscle layer that is opened/manipulated during the operative procedure AND patient has at least one of the following:

a) purulent drainage from a drain that is placed into the organ /space

b) organisms are identified from an aseptically obtained fluid or tissue in the organ/space by a culture or non-culture based microbiological method which is performed for diagnosis or treatment

c) an abscess or other evidence of infection involving the organ/space is detected during gross anatomical or histopathological exam or imaging test.

AND patients meets at least one criterion for a specific organ/space infection site infection (Urinary system infection and Intraabdominal infection for patients undergoing radical cystectomy, defined according to CDC surveillance definitions 2017)

Urinary tract infections will be classified as follows (20):

1). Symptomatic Urinary tract infection

Patient must meet 1, 2, and 3 below:

1. Patient had an indwelling urinary catheter that had been in place for > 2 days on the date of event (day of device placement = Day 1) AND was either:

- Present for any portion of the calendar day on the date of event†,
  OR
- Removed the day before the date of event‡

2. Patient has at least ***one*** of the following signs or symptoms:

• fever (>38.0°C)
• suprapubic tenderness
• costovertebral angle pain or tenderness
• urinary urgency ^
• urinary frequency ^
• dysuria ^

3. Patient has a urine culture with no more than two species of organisms identified, at least one of which is a bacterium of ≥105 CFU/ml (See Comments). All elements of the UTI criterion must occur during the Infection Window Period (See Definition Chapter 2 Identifying HAIs in NHSN).

2). Asysmptomatic bacteremic urinary tract infection

Patient must meet 1, 2, and 3 below:

1. Patient with***** or without an indwelling urinary catheter has no signs or symptoms of SUTI

2. Patient has a urine culture with no more than two species of organisms identified, at least one of which is a bacterium of ≥105 CFU/ml

3. Patient has organism identified from blood specimen with at least ***one*** matching bacterium to the bacterium identified in the urine specimen.

*****Patient had an indwelling urinary catheter in place for >2 calendar days on the date of event, with day of device placement being Day 1, and catheter was in place on the date of event or the day before.
** Organisms identified by a culture or non-culture based microbiologic testing method which is performed for purposes of clinical diagnosis or treatment

5.2 Secondary Outcomes

The following secondary outcomes will be assessed

- Rates of antibiotic-associated adverse events (Direct toxicity (renal, hepatic, hematological), gastrointestinal AEs, hypersensitivity)
- Rate of *C.difficile* colitis will be assessed
- Crude mortality in patients receiving short-term vs. extended antibiotic prophylaxis
- Frequency of (multi-) resistant bacteria in subsequent, routinely collected urine samples
- Directly antibiotic-associated costs incurred during short-term vs. extended antibiotic prophylaxis (assessed at day 30).
- Changes in the fecal microflora after short-term vs. extended PAP

5.3 Other Outcomes of Interest

Not applicable

5.4 Safety Outcomes

The primary study endpoint (rate/incidence of SSI) is the main safety objective. In addition the following safety objectives will be assessed in the two study arms:

- rates of antibiotic-associated adverse events (direct organotoxicity, gastrointestinal side effects, hypersensitivity reactions, indirect AEs such as line-associated inflammation/Infection)
- rate of C. difficile colitis
- crude mortality.
- Frequency of (multi-) resistant bacteria in subsequent urine samples.

6. STUDY DESIGN

6.1 General study design and justification of design

This is a prospective randomized non-inferiority trial comparing the efficacy of 24h perioperative antibiotic prophylaxis in patients undergoing radical cystectomy compared to the currently used extended (>48h) perioperative antibiotic prophylaxis.

All patient undergoing radical cystectomy at the Bern University Hospital will be eligible to participate in this trial. Based on sample size calculation we expect to include 98 patients per group, allowing to detect an effect difference of 5% and taking into account a post-randomization drop out rate of 7%. Based on a mean frequency of 79 radical cystectomies performed annually (2010-2015) a study duration of three years is projected.

The expected duration of study participation starting at baseline including follow-up is 90 days

Informed consent and Screening:

Patients will receive the study information and Patient informed consent form at the time point of their pre-surgery consultation. Patients who consent to participate in this study will undergo randomization at the day of surgery. Allocation of eligible patients will be performed by concealed block randomization (computer based), stratified for age and gender, and equal proportions will be assigned to the control (extended PAP) and intervention (24h PAP) group.

Intervention: Perioperative antibiotic prophylaxis

PAP will be administered as follows:

Substance	Dose/day	Duration	
		24h PAP	Extended PAP
Tobramycin	120mg iv single dose pre-surgery	x	x
Tobramycin	80mg iv q8h	24h	48h
Metronidazole	1000mg single dose pre-surgery	x	x
Metronidazole	500mg iv q12h	24h	48h
Amoxicillin/Clavulanate	1.2g/iv q8h	24h	Until all catheter/stentss are removed
Dosages will be adapted to renal functions and -if indicated- to patients weight; renal and neural toxicities will be monitored<br>In patients allergic to penicillin or beta lactam antibiotics, amoxicillin/clavulanate will be replaced by a combination of vancomycin (2x1g/24h, or adapted to creatinine clearance) plus ciprofloxacin 500mg<br>In patients colonized with resistant bacteria prior to surgery, the antibiotic regimen will be adapted according to the documented antibiotic sensitivities.<br>The first dose of antibiotics will be administered within 1 hour before incision.<br>Antibiotics will be stopped 24h post-surgery in the 24h PAP-group and after >48h (when all catheter/stents are removed) in the extended PAP group. A study nurse will call the treating team after 24h to ensure adherence to protocol (explicit question to treating physician: “did you stop the perioperative prophylaxis”) for the short-term group.			

Outcome assessment:

Primary outcome

Rate and incidence of surgical site infections in both treatment groups: Occurrence of surgical site infections according to current definitions ((CDC Procedure associated Module, Surgical Site Infection Event, January 2017) will be assessed at day 10, 30 and day 90 post-surgery by clinical and laboratory assessments. In addition to the event rate, the time point of SSI diagnosis will be recorded to allow for later time to event analysis.

Secondary outcomes

Rates of antibiotic-associated adverse events: antibiotic related AEs (clinical and laboratory events) will be recorded in the CRF at predefined time-points and assessed at the end of the study period

Rate of *C.difficile* colitis: in patients with diarrhea stool diagnostic to detect toxin secreting *C.difficile* will be performed according to current clinical practice, both diarrhea and positive results for *C.difficile* will be recorded in the CRF and rates will be calculated for both study groups. Also, any treatment initiated for suspected or proven *C.difficile* colitis will be recorded in the CRF.

Crude mortality at day 30 and day 90 post surgery in patients receiving short-term vs. extended antibiotic prophylaxis (aim 2) will be calculated.

Frequency of (multi-) resistant bacteria in patients receiving short-term vs. extended antibiotic prophylaxis will be analyzed in urine samples routinely collected according to current clinical practice

Directly antibiotic-associated costs incurred during short-term vs. extended antibiotic prophylaxis (aim 4) will be assessed at day 30 based on financial information provided by the hospital Department manager.

Fecal samples will be collected at day 0, day 3, 7 and 14 and 30. Changes in the fecal microflora after short-term vs. extended PAP will be analysed by PCR methods at a later time point (aim 3)

6.2 Methods of minimising bias

6.2.1 Randomisation

Allocation of eligible patients will be performed by concealed block randomization (computer based), stratified for age and gender, and equal proportions will be assigned to the control (extended PAP) and intervention (24h PAP) group.

6.2.2 Blinding procedures

No blinding procedures are planned for this study

6.2.3 Other methods of minimising bias

N/A

6.3 Unblinding Procedures (Code break)

N/A (The interventional part of the study will be conducted in an unblinded fashion)

7. STUDY POPULATION

7.1 Eligibility criteria

Participants fulfilling all of the following inclusion criteria are eligible for the study, for example:

- Informed Consent as documented by signature
- Radical cystectomy scheduled at Department for Urology, Bern University Hospital
- Age>18y

The presence of any one of the following exclusion criteria will lead to exclusion of the participant:

- Contraindications to the class of drugs under study, e.g., known hypersensitivity or allergy to class of drugs including alternatives described in the protocol or the investigational product,
- Women who are pregnant or breast feeding,
- Inability to follow the procedures of the study, e.g., due to language problems, psychological disorders, dementia, etc. of the participant,
- Enrolment of the investigator, his/her family members, employees and other dependent persons,

7.2 Recruitment and screening

Patients scheduled to undergo radical cystectomy at the Urology Clinic of Bern will receive the study information during the pre-surgery visit. Recruitment/Signing of PIC and screening will be done during the pre-surgery visit by the anesthesiologist in charge. No payments or compensations are planned for study participants.

7.3 Assignment to study groups

Allocation of eligible patients to the two study arms will be performed by concealed block randomization (computer based), stratified for age and gender, and equal proportions will be assigned to the control (extended PAP) and intervention (24h PAP) group. Patients will be randomized on the day of the scheduled surgery and assigned to their allocated treatment by the anesthesiologist in charge..

7.4 Criteria for withdrawal / discontinuation of participants

The participant can withdraw from the study at any time-point, without the need to give a reason to the investigators. The subject has to be withdrawn from the study prior the expected completion if there is a failure of the subject to adhere to the protocol requirements.

All subjects experiencing a serious adverse event considered to be related to the study intervention should be withdrawn from the study. Primary endpoint events (surgical site infections and urinary tract infections) are exempted from this rule.

Reasons for discontinuation from the clinical trial will be documented on the Study Termination Form.

Allergic reaction to antibiotics: In patients who develop an allergic reaction that is likely to be related to or caused by one of the prescribed antibiotics the study intervention will be modified as follows: Switching to a different substance class will not represent a protocol violation however premature cessation of antibiotics will be a protocol violation in the long term-group.

Active bacterial infection: Extended prescription of antibiotics due to an active bacterial infection diagnosed during the intervention period will not represent a protocol violation but will have to be carefully documented for later analysis.

8. STUDY INTERVENTION

8.1 Identity of Investigational Products (treatment / medical device)

Eligible patients will be randomized in equal proportions between the standard (extended duration) and experimental /investigative (short-term) PAP group, and will receive a perioperative prophylaxis for either 24h or for an extended course of >48h (until all catheter/stents are removed)

The parenteral antibiotic regime used will be the same for the two groups and consists of a combination therapy using

- **Extended PAP (>48h, Standard procedure at Department of Urology, University Hospital Bern)**

Tobramycin: 3 x 80 mg i.v. or adapted to creatinine clearance for 48h

Metronidazole: loading dose preoperatively 1000 mg, then 2 x 500 mg i.v. for 48h

Augmentin: 3 x 1.2 gr i.v. until all catheter/stents/drains removed

- **24h PAP (24h, in adherence to current guideline recommendations)**

Tobramycin: 3 x 80 mg i.v. or adapted to creatinine clearance for 24h

Metronidazole: loading dose preoperatively 1000 mg, then 2 x 500 mg i.v. for 24h

Augmentin: 3 x 1.2 gr i.v. for 24h

In patients allergic to penicillins or betalactam antibiotics, amoxicillin/clavulanate will be replaced with a combination of Vancomycin (2x1g/24h, or adapted to creatinine clearance) plus Ciprofloxacin 500mg according to current standard practice

In patients colonized with resistant bacteria prior to surgery, the antibiotic regime will be adapted according to the documented antibiotic sensitivities

The first dose of antibiotics will be applied within 1 hour before incision.

Antibiotics will be stopped 24h post-surgery in the 24h PAP-group and after >48h in the extended PAP group.

For patients in the short term group a study nurse will call the treating team after 24h to ensure adherence to protocol (question to treating physician: "did you stop the perioperative prophylaxis").

8.1.1 Experimental Intervention (treatment / medical device)

24h PAP (24h, in adherence to current guideline recommendations)
Tobramycin: 3 x 80 mg i.v. or adapted to creatinine clearance for 24h
Metronidazole: loading dose preoperatively 1000 mg, then 2 x 500 mg i.v. for 24h
Augmentin: 3 x 1.2 gr i.v. for 24h

In patients allergic to penicillins or betalactam antibiotics, amoxicillin/clavulanate will be replaced with a combination of Vancomycin (2x1g/24h, or adapted to creatinine clearance) plus Ciprofloxacin 500mg

Antibiotics will be stopped 24h post-surgery in the 24h PAP-group and after >48h in the long-term PAP group. For patients in the short term group a study nurse will call the treating team after 24h to ensure adherence to protocol (question to treating physician: "did you stop the perioperative prophylaxis").

8.1.2 Control Intervention (standard/routine/comparator treatment / medical device)

Long-term PAP (>48h, Standard procedure at Department of Urology, University Hospital Bern
Tobramycin: 3 x 80 mg i.v. or adapted to creatinine clearance for 48h

Metronidazole: loading dose preoperatively 1000 mg, then 2 x 500 mg i.v. for 48h

Augmentin: 3 x 1.2 gr i.v. until all catheter/stents/drains removed

8.1.3 Packaging, Labelling and Supply (re-supply)

No special packaging, labelling (no investigational products)

8.1.4 Storage Conditions

Supply, storage and return of medical products used in this study (antibiotics) will be handled according to hospital standard procedures

8.2 Administration of experimental and control interventions

8.2.1 Experimental Intervention

Perioperative antibiotic prophylaxis will be administered within 1 hour before incision as per standard procedure with no difference in drug regimens between the experimental and control group.

24h PAP

**Substance**	**Dose/route/frequency**	**Duration**
Tobramycin	120mg iv single dose pre-surgery	x
Tobramycin	80mg iv q8h	24h
Metronidazole	1000mg single dose pre-surgery	x
Metronidazole	500mg iv q12h	24h
Amoxicillin/Clavulanate	1.2g/iv q8h	24h
Dosages will be adapted to renal functions and -if indicated- to patients weight; renal and neural toxicities will be monitored<br>In patients allergic to penicillin or beta lactam antibiotics, amoxicillin/clavulanate will be replaced by a combination of vancomycin (2x1g/24h, or adapted to creatinine clearance) plus ciprofloxacin 500mg<br>In patients colonized with resistant bacteria prior to surgery, the antibiotic regimen will be adapted according to the documented antibiotic sensitivities.<br>The first dose of antibiotics will be administered within 1 hour before incision.<br>Antibiotics will be stopped 24h post-surgery in the 24h PAP-group. A study nurse will call the treating team after 24h to ensure adherence to protocol (explicit question to treating physician: “did you stop the perioperative prophylaxis”) for the short-term group.		

8.2.2 Control Intervention

Perioperative antibiotic prophylaxis will be administered within 1 hour before incision as per standard procedure with no difference in drug regimens between the experimental and control group.

Extended PAP

**Substance**	**Dose/route/frequency**	**Duration**
Tobramycin	120mg iv single dose pre-surgery	x
Tobramycin	80mg iv q8h	48h
Metronidazole	1000mg single dose pre-surgery	x
Metronidazole	500mg iv q12h	48h
Amoxicillin/Clavulanate	1.2g/iv q8h	Until all catheter/stents are removed
Dosages will be adapted to renal functions and -if indicated- to patients weight; renal and neural toxicities will be monitored<br>In patients allergic to penicillin or beta lactam antibiotics, amoxicillin/clavulanate will be replaced by a combination of vancomycin (2x1g/24h, or adapted to creatinine clearance) plus ciprofloxacin 500mg<br>In patients colonized with resistant bacteria prior to surgery, the antibiotic regimen will be adapted according to the documented antibiotic sensitivities.<br>The first dose of antibiotics will be administered within 1 hour before incision.<br>Antibiotics will be stopped after >48h (when all catheter/stents are removed) in the extended PAP group.		

8.3 Dose / Device modifications

Dosages will be adapted to renal functions and -if indicated- to patient weight; renal and ontological toxicities will be monitored according to current standard procedures

In patients allergic to penicillin or betalactam antibiotics, amoxicillin/clavulanate will be replaced by a combination of vancomycin (2x1g/24h, or adapted to creatinine clearance) plus ciprofloxacin (currend standrard practice)

In patients colonized with resistant bacteria prior to surgery, the antibiotic regimen will be adapted according to the documented antibiotic sensitivities (standard procedures).

8.4 Compliance with study intervention

A study nurse will call the treating team after 24h to ensure adherence to protocol (question to treating physician: “did you stop the perioperative prophylaxis?”) for the short term group.

Allergic reaction to antibiotics: In patients who develop an allergic reaction likely to be related to or caused by one of the prescribed antibiotics, the study intervention will be modified as follows: Switching to a different substance class will not represent a protocol violation, however premature cessation of antibiotics will be a protocol violation in the extended PAP group

8.5 Data Collection and Follow-up for withdrawn participants

Anonymization of data collected during this study will not be possible and patients are adequately informed of this fact prior to consenting to the trial (KlinV Art 9).

The following encoded data and biological samples collected prior to the timepoint of withdrawal will be used for analysis.:

- medical records
- Laboratory data
- Microbiological samples (stool and urine)
- Clinical data

8.5.1.1.1 **Art. 9** Consequences of revocation of consent

[1] If consent is revoked, the biological material and health-related personal data of the person concerned must be anonymised after data evaluation has been completed.

[2] Anonymisation of the biological material and personal data may be dispensed with if:

a.

the person concerned expressly renounces this right when revoking consent; or

b.

[3] Persons revoking consent must be offered any follow-up care required to protect their health.

8.6 Trial specific preventive measures

Not applicable.

8.7 Concomitant Interventions (treatments)

Any concomitant care and intervention compliant with current standards of care are permitted during the study period. Co-medications will be recorded in the CRF

8.8 Study Drug / Medical Device Accountability

NA (standard procedures)

8.9 Return or Destruction of Study Drug / Medical Device

NA (standard procedures)

STUDY SCHEDULE

9. STUDY ASSESSMENTS

9.1 Study flow chart(s) / table of study procedures and assessments

TIMEPOINT	Screening	Baseline (surgery)	T1	T2	T3	T4	T5	T6	T7	T8
Day/h	-10d - -1d	0	1d 24h	2d 48h	3d 72h	5d	7d	10d (+4d)	30d (+7d)	90d (+/-7d)
SCREENING/BASELINE										
Patient information and informed consent	x									
Inclusion/Exclusion criteria	x									
Demographic data[1)]	x									
Medical history [2)]	x									
Colonization with MDR bacteria?	x									
Concommitant medication	x	x								
Physical examination (T, BD, HR, weight, height)	x	x								
Blood examination [3)]	x	x								
Urine examination [4)]										
Microbiology [5)]		x								
Stool sample	x									
Randomization/Allocation		x								
Adverse Events	x	x								
INTERVENTION										
Short term PAP [6)]			***							
Extended PAP [7)]										
Outcome ASSESSMENT										
SSI [8)]					x	x	x	x	x	x
Adverse Events [10)]			x	x	x	x	x	x	x	x
Clinical outcome variables [9)]			x	x	x	x	x	x	x	
Concomitant medication			x	x	x	x	x	x	x	
Physical examination (T, BD, HR, weight, height)			x	x	x	x	x	x	x	
Blood examination [3)]			x	x	x	x	x	x	x	
Urine examination [4)]			x	x	x	x	x	x	x	x
Microbiology [5)]	x				x		x	x	x	x
Stool sample	x							x	x	
Total days of antibiotics prescribed									x	x
Total days of prophylactic ABx									x	x
Costs of antibiotics									x	x
Number of inpatient days									x	x

*at pre-op assessment
***Tel study nurse (explicit question to treating physician: “did you stop the perioperative prophylaxis”)

1) age, gender, ethnicity, language
2) current Diagnosis, Diabetes, smoking, heart disease, hypertension, previous UTI
3) Hematogram V, CRP, Creatinine, Urea, ALAT ASAT
4) Urine chemistry
5) Results of urine culture
6) 24h prophylaxis
7)>48h prophylaxis (stop when all catheter/stents are removed)
8) signs of SSI (if yes further classified)
9) According to CRF: Vital signs (T, BP, HR) , signs of blood stream infection (yes/no), signs of UTI (yes/no),
10) signs of antibiotic-related AE (nausea, diarrhea, allergic reaction (skin rash or other), hepatotoxicity (ASAT, ALAT >2xULN), renal toxicity (Creatinine >150umol/L or >50% increase from baseline)) line associated infections? Diarrhea yes/no (if yes: Detection of C.difficile?)

9.2 Assessments of outcomes

9.2.1 Assessment of primary outcome

Primary outcome

Rate of Surgical site infections in both treatment groups: Occurrence of surgical site infections according to current definitions (see below) will be assessed at day 10, 30 and 90 post surgery by clinical and laboratory assessments. In addition to the event rate, the time point of SSI diagnosis will be recorded to allow for later time-to-event analysis.

Surgical site infections (SSI) will be diagnosed as defined by the Centers for Disease Control and Prevention criteria (CDC Procedure associated Module, Surgical Site Infection Event, January 2017).

Occurence of primary outcome events will be assessed in a structured manner at defined time points as depicted in the study flow chart. Start date and stop date of SSI as well as category of SSI will be noted.

9.2.2 Assessment of secondary outcomes

Secondary outcomes

Rates of antibiotic-associated adverse events: antibiotic related AEs (clinical and laboratory events)will be recorded in the CRF at predefined time-points and assessed at the end of the study period.

Rate of C.difficile colitis: in patients with diarrhea stool diagnostics to detect toxin secreting C.difficile will be performed according to current clinical practice, both diarrhea and positive results for C.difficile will be recorded in the CRF and rates will be calculated for both study groups at the end of the study period (d 30)

Crude mortality at day 30and 90 post surgery in patients receiving short-term vs. extended antibiotic prophylaxis (aim 2) will be calculated.

Frequency and type of (multi-) resistant bacteria in patients receiving short-term vs. extended antibiotic prophylaxis will be analysed in urine samples routinely collected according to current clinical practice (cumulative incidence per patient at day 30)

Directly antibiotic-associated costs incurred during short-term vs. extended antibiotic prophylaxis (aim 4) will be assessed at day 30 based on financial information provided by the hospital.

9.2.3 Assessment of other outcomes of interest

In patients consenting to the storage and further analysis of biological material, fecal samples will be collected at day 0 and day 14. Aliquots of samples will be frozen and stored at -20°C until further analysis. Changes in the fecal microflora after short-term vs. extended PAP will be analysed by PCR comparing intraindividual variance (comparing day 0 vs day 14).

9.2.4 Assessment of safety outcomes

9.2.4.1 Adverse events

At every study visit/assessment time point, we will record adverse event information in the eCRF (i.e., type, category, time of onset, duration, resolution, action to be taken, assessment of intensity, relationship with the study treatment). For the definition and procedures we are referring to section 10.

9.2.4.2 Laboratory parameters

Safety laboratory analyses will be assessed and recorded in the CRF at predefined time points. The analyses will be done at the laboratories of the participating centers according to hospital routine processes. Laboratory AEs will be defined as previously described (21)

9.2.4.3 <u>Vital signs</u>

Vital signs (i.e., blood pressure, heart rate, temperature) are assessed regularly according to current clinical practice of postoperative patients care at the participating centers

9.2.5 Assessments in participants who prematurely stop the study

Follow-up procedures and assessments in participants who are withdrawn from the study prematurely (e.g., recording of adverse events, physical examination, laboratory parameters, vital signs) will follow standard clinical practice: These patients will continue to be monitored for signs of SSI, however no information will be used within the study framework after the time of withdrawal. The follow up period will be according to standard practice

9.3 Procedures at each visit

9.3.1 Screening visit (Day -10 to -0)

- Obtain written informed consent
- Check inclusion and exclusion criteria
- Obtain demographics and medical history
- Assess concomitant medication
- Perform a physical examination and obtain vital signs
- Obtain routine laboratory measurements (i.e., full blood count, creatinine, ASAT, ALAT, alkaline phosphatase, bilirubin and urine analysis)
- Urine culture
- Adverse event assessment (Log sheet)
- Collect stool sample for patients who agree to provide biological samples for later analysis

9.3.2 Baseline visit, Day 0, day of surgery (T0)

- Allocate to study arm according to randomization
- Note Performed surgery
- Start perioperative prophylaxis according to randomization
- Obtain vital signs
- Obtain routine laboratory measurements (i.e., full blood count, creatinine, ASAT, ALAT, alkaline phosphatase, bilirubin and urine analysis)
- Assess concomitant medication
- Urine culture
- Assess for urinary tract infection/asymptomatic bacteriuria
- Log catheter placements
- Adverse event assessment

9.3.3 Follow-up visits and assessments

Day 1 postsurgery (T1)

- Continue or stop PAP according to randomization
- Obtain vital signs
- Obtain routine laboratory measurements (i.e., full blood count, creatinine, ASAT, ALAT, alkaline phosphatase, bilirubin and urine analysis)

- Assess concomitant medication
- Log catheters
- Adverse event assessment
- Study nurse will call 24-36h postsurgery to ensure cessation of PAP in short term group

Day 2 postsurgery (T2)

- Check if PAP has been stopped in short term arm
- Record continuation/cessation of PAP in long-term arm
- Obtain vital signs
- Obtain routine laboratory measurements (i.e., full blood count, creatinine, ASAT, ALAT, alkaline phosphatase, bilirubin and urine analysis)
- Assess concomitant medication
- Assess and log catheters in situ
- Adverse event assessment

Day 3 postsurgery (T3)

- Record continuation/Cessation of PAP in long-term arm
- Obtain vital signs
- Assess for presence of SSI and UTI
- Obtain routine laboratory measurements (i.e., full blood count, creatinine, ASAT, ALAT, alkaline phosphatase, bilirubin and urine analysis)
- Obtain urine culture
- Assess concomitant medication
- Assess and log catheters in situ
- Adverse event assessment
-

Day 5 postsurgery (T4)

- Record continuation/Cessation of PAP in long-term arm
- Obtain vital signs
- Assess for presence of SSI and UTI
- Obtain routine laboratory measurements (i.e., full blood count, creatinine, ASAT, ALAT, alkaline phosphatase, bilirubin and urine analysis)
- Assess concomitant medication
- Assess and log catheters in situ
- Adverse event assessment

Day 7 postsurgery (+/-1d) (T5)

- Record continuation/cessation of PAP in long-term arm
- Obtain vital signs
- Assess for presence of SSI and UTI
- Obtain routine laboratory measurements (i.e., full blood count, creatinine, ASAT, ALAT, alkaline phosphatase, bilirubin and urine analysis)
- Obtain urine culture
- Assess concomitant medication
- Assess and log catheters in situ
- Adverse event assessment

- ***Day 10 postsurgery (+4d) (T6)***

- Record continuation/Cessation of PAP in long-term arm
- Obtain vital signs
- Assess for presence of SSI and UTI
- Obtain routine laboratory measurements (i.e., full blood count, creatinine, ASAT, ALAT, alkaline phosphatase, bilirubin and urine analysis)
- Obtain urine culture
- Assess and log catheters in situ
- Assess concomitant medication
- Adverse event assessment
- Collect stool sample

Day 30 postsurgery (+4d) (T7)

- Record continuation/Cessation of PAP in long-term arm
- Obtain vital signs
- Assess for presence of SSI and UTI
- Obtain routine laboratory measurements (i.e., full blood count, creatinine, ASAT, ALAT, alkaline phosphatase, bilirubin and urine analysis)
- Obtain urine culture
- Assess concomitant medication
- Adverse event assessment
- Collect stool sample Assess total days of anibiotics prescribed
- Assess total days of PAP prescribed
- Costs of antibiotics
- Costs of PAP
- Number of inpatient days
-
-
- **9.3.4. Final visit, Day 90 post PAP (T8)**
- Record continuation/or time point of cessation of PAP in long-term arm
- Obtain vital signs
- Assess for presence of SSI and UTI
- Obtain routine laboratory measurements (i.e., full blood count, creatinine, ASAT, ALAT, alkaline phosphatase, bilirubin and urine analysis)
- Assess concomitant medication
- Adverse event assessment
- sample
- Assess total days of anibiotics prescribed
- Assess total days of PAP prescribed
- Costs of antibiotics
- Costs of PAP
- Number of inpatient days

10. SAFETY

10.1 Drug studies

During the entire duration of the study, all adverse events (AE) and all serious adverse events (SAEs) are collected, fully investigated and documented in source documents and case report forms (CRF). Study duration encompassed the time from when the participant signs the informed consent until the last protocol-specific procedure has been completed, including a safety follow-up period. **Art. 63** Documentation and notification of serious adverse events

[1] If, in the course of a clinical trial, serious adverse events occur in participants in Switzerland, and it cannot be excluded that the events are attributable to the intervention under investigation, the investigator must document them in a standardised manner. In addition, the investigator shall report these events:

a.

to the sponsor within 24 hours after they become known; and

b.

to the responsible ethics committee within 15 days.

[2] A serious adverse event is defined as any event which:

a.

requires inpatient treatment not envisaged in the protocol or extends a current hospital stay;

b.

results in permanent or significant incapacity or disability;

c.

is life-threatening or results in death; or

d.

causes a congenital anomaly or birth defect.

[3] If necessary in order to guarantee participants' safety and health, further adverse events which must be documented or reported are to be designated in the protocol or at the request of the responsible ethics committee.

[4] If, in the case of a multicentre clinical trial, serious adverse events occur at one of the trial sites, the coordinating investigator shall also report the events as specified in paragraphs 1 and 3 to the responsible ethics committee concerned, within the same period.[1]

---

[1] Correction of 27 Dec. 2013 (AS **2013** 5579).

10.1.1 Definition and assessment of (serious) adverse events and other safety related events

An **Adverse Event (AE)** is any untoward medical occurrence in a patient or a clinical investigation participant administered a pharmaceutical product and which does not necessarily have a causal relationship with the study procedure. An AE can therefore be any unfavourable and unintended sign (including an abnormal laboratory finding), symptom, or disease temporally associated with the use of a medicinal (investigational) product, whether or not related to the medicinal (investigational) product. [ICH E6 1.2]

A **Serious Adverse Event (SAE)** is classified as any untoward medical occurrence that:

- results in death,
- is life-threatening,
- requires in-patient hospitalization or prolongation of existing hospitalisation,
- results in persistent or significant disability/incapacity, or
- is a congenital anomaly/birth defect.

In addition, important medical events that may not be immediately life-threatening or result in death, or require hospitalisation, but may jeopardise the patient or may require intervention to prevent one of the other outcomes listed above should also usually be considered serious. [ICH E2A]

Examples of such events are intensive treatment in an emergency room or at home for allergic bronchospasm, blood dyscrasias or convulsions that do not result in hospitalisation, or development of drug dependency or drug abuse.

SAEs should be followed until resolution or stabilisation. Participants with ongoing SAEs at study termination (including safety visit) will be further followed up until recovery or until stabilisation of the disease after termination.

Assessment of Causality

Both Investigator and Sponsor-investigator make a causality assessment of the event to the study drug, based on the criteria listed in the ICH E2A guidelines:

Relationship	Description
Definitely	Temporal relationship<br>Improvement after dechallenge*<br>Recurrence after rechallenge<br>(or other proof of drug cause)
Probably	Temporal relationship<br>Improvement after dechallenge<br>No other cause evident
Possibly	Temporal relationship<br>Other cause possible
Unlikely	Any assessable reaction that does not fulfil the above conditions
Not related	Causal relationship can be ruled out
*Improvement after dechallenge only taken into consideration, if applicable to reaction	

Note that other categories can be used. However, a definition has to be provided in the protocol.

Unexpected Adverse Drug Reaction

An “unexpected” adverse drug reaction is an adverse reaction, the nature or severity of which is not consistent with the applicable product information (e.g. Investigator’s Brochure for drugs that are not yet approved and Product Information for approved drugs, respectively). [ICH E2A]

Suspected Unexpected Serious Adverse Reactions (SUSARs)

The Sponsor-Investigator evaluates any SAE that has been reported regarding seriousness, causality and expectedness. If the event is related to the investigational product and is both serious and unexpected, it is classified as a SUSAR.

Note: In case of double-blinded studies, unblinding is needed in order to determine a SUSAR.

Assessment of Severity

Describe the severity grading scale in use for this study, depending on the type of study and disease, the grades for severity described in the “Common Terminology Criteria for Adverse Events CTCAE Version x” terminology may be used and should be referred to here. Other definitions and grades are possible and shall be provided in the protocol (e.g. grading scale with explanation or reference to source).

10.1.2 Reporting of serious adverse events (SAE) and other safety related events

Reporting of SAEs

All SAEs must be reported immediately and within a maximum of 24 hours to the Sponsor-Investigator of the study. The Sponsor-Investigator will re-evaluate the SAE and return the form to the site.

SAEs resulting in death are reported to the local Ethics Committee (via local Investigator) within 7 days.

Reporting of SUSARs

A SUSAR needs to be reported to the local Ethics Committee (local event via local Investigator) within 7 days, if the event is fatal, or within 15 days (all other events).

Reporting of Safety Signals

All suspected new risks and relevant new aspects of known adverse reactions that require safety-related measures, i.e. so called safety signals, must be reported to the Sponsor-Investigator within 24 hours. The Sponsor-Investigator must report the safety signals within 7 days to the local Ethics Committee (local event via local Investigator).

.

10.1.3 Follow up of (Serious) Adverse Events

For a Grade 4 clinical event or clinically significant Grade 4 laboratory abnormality confirmed by repeat testing that is considered related to investigational medical approach, investigational medical approach should be permanently discontinued and the subject managed according to local practice. The subject should be followed as clinically indicated until the laboratory abnormality returns to baseline or is otherwise explained, whichever occurs first.

Participants terminating the study (either regularly or prematurely) with reported ongoing SAE, or any ongoing AEs of laboratory values or of vital signs being beyond the alert will return for a follow-up investigation. This visit will take place up to 90 days after terminating the treatment period. Follow-up information on the outcome will be recorded on the respective AE page in the eCRF. All other information has to be documented in the source documents. Source data has to be available upon request.

In case a participant is lost to follow-up, efforts will be made and documented to contact the participant in order to encourage him/her to continue study participation as scheduled. In case of minor AE, a phone call to the participants may be acceptable. All new SAE that the investigators will be notified of within 30 days after discontinuation of study medication have to be reported in appropriate report forms and in the eCRF if required.

Follow-up investigations may also be necessary according to the investigator’s medical judgment even if the participant has no AE at the end of the study. However, information related to these investigations does not have to be documented in the eCRF but must be noted in the source documents.

11. STATISTICAL METHODS

11.1 Hypothesis

The efficacy and safety of 24h PAP (≤24h) vs. extended (>48h, until all catheter/stents removed) PAP will be compared in a randomized controlled clinical non-inferiority trial.

We define the proportion of surgical site infections (SSI) after 30 days in the control group (>48h PAP) to be pC and the proportion of SSI for shorter period (≤ 24h) pE. The null hypothesis is defined as the 95% confidence for the difference in rates (pC - pE) being within the interval of therapeutic equivalence (D). Formally, H0: (pC - pE) > D and H1: (pC - pE) < D.

11.2 Determination of Sample Size

Formally, H0: (pC - pE) > D and H1: (pC - pE) < D. The margin D was defined based on clinical and statistical reasoning: The local rate of SSIs at the Urology Clinic with currently >48h days of PAP is not known. Based on available literature, we assumed a baseline SSI rate of 10% with standard treatment and an increased risk of 15% SSI in the intervention group, with the difference being approximately 5%. Due to the large variation in reported SSI incidence we define the interval of therapeutic difference to be 2 times the difference found by Hara et al (ref), i.e., D = 0.07 (93 patients/group). Taking into account a reported post-randomization dropout rate of 7% (6/83), we dimensioned the sample size for the per-protocol analysis including this inflation factor to be 186/(1-0.07) = 196 patients with 98 patients in each group. Based on a mean frequency of 79 radical cystectomies performed annually (2010-2015) a study duration of three years is projected.

11.3 Statistical criteria of termination of trial

The trial will be terminated at the time point when the last participant has had his 30 FUP day visit.

There is no planned stopping rule for the trial.

11.4 Planned Analyses

Descriptive statistics will be produced based on mean and standard deviation for continuous, normally distributed variables; median and inter-quartile range for continuous, non-normally distributed variables; and percentages of the appropriate marginal totals for dichotomous and other categorical variables. The Shapiro-Wilk test will be used to investigate departures from normality for continuous variables.

Group differences will be tested using the t-test or analysis of variance methods for continuous, normally distributed variables, the Wilcoxon-Mann-Whitney or Kruskal-Wallis tests for continuous, non-normally distributed variables, and the chi-square (respectively proportions) test for dichotomous and categorical variables. Wald-type confidence intervals will be used for dichotomous and categorical variables.

A significance level of 5% will used throughout the study, Bonferroni corrected for multiple testing where appropriate

11.4.1 Datasets to be analysed, analysis populations

Populations:

24h PAP, extended PAP

Analysis:

Both, intention to treat analysis (two groups as per randomisation) and per protocol analysis (two

groups as per completed protocol) will be performed in order to confirm or reject non-inferiotity of the 24h PAP compared to the extended PAP.

Although risk stratification will be performed at randomization we plan to adjust outcome comparisons between the two groups for confounding factors. Should the results point to different outcomes in specific subgroups (ie presence of certain risk factors) these subgroups will be further investigated.

11.4.2 Primary Analysis

Describe the intended primary analysis that will be done, when and how and by whom it will be done, The primary study outcome (incidence and rate of SSI in both groups) will be assessed after 70 patients have been recruited (interim analysis), and again upon completion of the study (recruitment and study completion of 140 patients)

11.4.3 Secondary Analyses

- Rates of antibiotic-associated adverse events will be assessed for the two populations
- Rate of C.difficile colitis per patient will be calculated
- Crude mortality at day 30 and day 90 post surgery in patients receiving short-term vs. extended antibiotic prophylaxis (aim 2) will be calculated.
- Frequency of (multi-) resistant bacteria in urine samples patients receiving short-term vs. extended antibiotic prophylaxis will be analysed
- Directly antibiotic-associated costs incurred during short-term vs. extended antibiotic prophylaxis (aim 4) will be assessed at day 30 based on financial information provided by the hospital
- In patients consenting to the collection of biological samples, fecal samples will be collected at day 0, day 14, 30 and 90. Changes in the fecal microflora after short-term vs. extended PAP will be analysed by PCR methods at a later time point

11.4.4 Interim analyses

An interim analysis of the primary end-point will be performed after recruitment of 70 (35 patients in each arm) and again after recruitment of 140 patients (70 patients /arm).

The purpose of this analysis is to assess the safety/ non-inferiority of the 24h PAP. Should there be evidence for a higher SSI incidence in patients receiving 24h PAP not compatible with non-inferiority criteria, study termination would have to be considered.

11.4.5 Safety analysis

The main safety concern of this study lies with the possibility of a higher SSI incidence in the 24h PAP group. Assessment of the rate/incidence of SSI is the primary study outcome and will be assessed as such.

In addition rate/incidence of antibiotic associated adverse events including rates of C.difficile colitis will be assessed as secondary endpoints.

11.4.6 Deviation(s) from the original statistical plan

Not applicable

11.5 Handling of missing data and drop-outs

Missing values and drop- outs will be calculated as events (i.e. SSI) in the ITT analysis

12. QUALITY ASSURANCE AND CONTROL

The sponsor investigator is responsible to have written standard operating procedures (SOPs) and working instructions (WIs) in place for the study and provide those to the participating study sites. The site PI must have SOPs and WIs relevant for the study available and are responsible for proper traing of all involved study personnel for the respective procedures.

Monitoring and maybe Audits will be conducted during the study for quality assurance purposes by the Sponsor-Investigator or a delegated person.

12.1 Data handling and record keeping / archiving

The study will strictly follow the protocol. If any changes become necessary they must be described in a protocol amendment which will have to be signed by the sponsor investigator and will need to be approved by the CEC.

12.1.1 Case Report Forms

The investigators will use electronic case report forms eCRF which will be filled out at each study time point/study visit for all participants. All screened patients (ie patients who entered the study, patients who did not enter the study due to ineligibility, eligible patients who were not enrolled) will have to be documented in a screening log. The investigator will document enrolled study participants in the enrolment log.

For data management, monitoring , reporting and coding an internet based secure data base (REDCap), developed in agreement with GCP guidelines will be used. It is the responsibility of the investigator to assure that all data will be entered completely and correctly in the data base

Corrections in the eCRF may only be done by the investigator or by other authorised persons. In case of corrections the original data entries will be archived in the system and can be made visible. For all data entries and correction dates, time of day and person who is performing the entries will be generated automatically.

eCRFs must be kept current to reflect participant status at each phase during the course of study. Participants must not to be identified in the eCRF by name. Appropriate coded identification must be used (e.g., initials will not be used in combination with the date of birth in the eCRF for identification of the study participant). It must be assured that any authorised person, who may perform data entries and changes in the eCRF, can be identified. A list with signatures and initials of all authorised persons will be filed out in the study site file and the trial master file, respectively.

Documented medical histories and narrative statements relative to the participant's progress during the study will be kept. These records will also include the following: originals or copies of laboratory and other medical test results (e.g. electrocardiograms, etc.) which must be kept on file with the individual participant's eCRF.

The investigators assure to perform a complete and accurate documentation of the participant data in the eCRF. All data entered into the eCRF with exception of data recorded in the electronic medical charts must also be available in the individual participant file either as print-outs or as notes taken by either the investigator or another responsible person assigned by the investigator.

Essential documents must be retained for at least 10 years after the regular end or a premature termination of the respective study (KlinV Art. 45). Any patient files and source data must be archived for the longest possible

12.1.2 Specification of source documents

The following documents are considered source data, including but not limited to:

- SAE worksheets
- Nurse records, records of clinical coordinators, doctors records
- Medical records from other department(s) or other hospital(s), discharge letters and correspondence with other departments/hospitals, if participant visited any during the study period and the post study period.
- Demographic data (age, sex, ethnicity)
- Inclusion and exclusion criteria details
- Participation in study and signed and dated Informed Consent Forms
- Visit dates

- Medical history and physical examination details
- Concomitant medication
- All adverse events
- Key efficacy and safety data (as specified in the protocol)
- Results of relevant examinations
- Laboratory print-outs or electronic records

Reason for premature discontinuation, if applicable

12.1.3 Record keeping / archiving

All study data must be archived for a minimum of 10 years after study termination or premature termination of the clinical trial. The main study data are stored in the archives of the study nurse's office at the Department of Infectious Disease's outpatient clinic at Inselspital, Bern

12.2 Data management

12.2.1 Data Management System

We will use an eCRF developed in REdCap®. Access to the data base will be limited to authorized persons including all staff members. Prior to the beginning of the study, the eCRF will be tested by two investigators at the study site

12.2.2 Data security, access and back-up

Access to study data is limited to some members of this trial and stated in the separate document including all staff members. Access to data will only be possible by entering a password.

12.2.3 Analysis and archiving

All data obtained within the current study will be archived for at least 10 years after termination of the study. All statistical analyses will be performed using Stata 13.0. or R

12.2.4 Electronic and central data validation

The monitoring team of the study will check and assure the quality of the collected data according to the monitoring plan

12.3 Monitoring

Monitoring visits prior to the start and during the course of the study will be performed by the monitoring team to help to follow the progress of the clinical study, to assure utmost accuracy of the data and to detect possible errors at an early time point. The Sponsor Investigator will organize professional independent monitoring for the study. Monitoring will be conducted according to the Monitoring Plan.

All original data including all patient files, progress notes and copies of laboratory and medical test results must be available for monitoring. The monitor will review all or a part of the eCRFs and written informed consents. The accuracy of the data will be verified by reviewing the above referenced documents.

12.4 Audits and Inspections

A quality assurance audit/inspection of this study may be conducted by the competent authority. The quality assurance auditor/inspector will have access to all medical records, the investigator's study related files and correspondence, and the informed consent documentation that is relevant to this clinical study.

The investigator will allow the persons being responsible for the audit or the inspection to have access

to the source data/documents and to answer any questions arising. All involved parties will keep the patient data strictly confidential.

12.5 Confidentiality, Data Protection

Direct access to source documents will be permitted for purposes of monitoring, audits and inspections. Access to the protocol, dataset, and statistical code during and after the study will include all members listed in this protocol.

12.6 Storage of biological material and related health data

All data obtained within the current study including additional laboratory measurements will be stored according to the separate protocols from the related laboratories. All participants will be asked in a separate written informed consent for storage of stool samples. In case a participant refuses the storage of stool samples, these samples will be destroyed. We refer to Swiss legal requirements and confirm that samples or genetic data are only stored and used for research purposes with the participants consent.

13. PUBLICATION AND DISSEMINATION POLICY

The results of the proposed study will be presented at international conferences and will be published in peer-reviewed international scientific journals.

The study-participants will be informed directly by their attending physician about the study results

14. FUNDING AND SUPPORT

14.1 Funding

Funding for this study is provided by an Insel Grant awarded to Dr, Thurnheer

14.2 Other Support

Not applicable

15. INSURANCE

Not applicable (category A study)

16. REFERENCES

- Declaration of Helsinki, Version October 2013, (http://www.wma.net/en/30publications/10policies/b3/index.html)
- International Conference on Harmonization (ICH, 1996) E6 Guideline for Good Clinical Practice. (http://www.ich.org/fileadmin/Public_Web_Site/ICH_Products/Guidelines/Efficacy/E6_R1/Step4/E6_R1__Guideline.pdf)
- International Conference on Harmonization (ICH, 1997) E8 Guideline: General Considerations for Clinical Trials http://www.ich.org/fileadmin/Public_Web_Site/ICH_Products/Guidelines/Efficacy/E8/Step4/E8_Guideline.pdf)
- Humanforschungsgesetz, HFG Bundesgesetz über die Forschung am Menschen (Bundesgesetz über die Forschung am Menschen, HFG) vom 30. September 2011/ Loi fédérale relative à la recherche sur l'être humain (loi relative à la recherche sur l'être humain, LRH) du 30 septembre 2011. (http://www.bag.admin.ch/themen/medizin/00701/00702/07558/index.html?lang=de)
- Verordnung über klinische Versuche in der Humanforschung (Verordnung über klinische Versuche, KlinV) vom 20. September 2013 / Ordonnance sur les essais cliniques dans le cadre de la recherche sur l'être humain (Ordonnance sur les essais cliniques, OClin) du 20 septembre 2013. (http://www.bag.admin.ch/themen/medizin/00701/00702/12310/index.html?lang=de)
- Heilmittelgesetz, HMG Bundesgesetz über Arzneimittel und Medizinprodukte (Heilmittelgesetz, HMG) vom 15. Dezember 2000/Loi fédérale sur les médicaments et les dispositifs médicaux (Loi sur les produits thérapeutiques, LPT) du 15 décembre 2000. (http://www.admin.ch/ch/d/sr/8/812.21.de.pdf)
- ISO 14155:2011 Clinical investigation of medical devices for human subjects -- Good clinical practice (www.iso.org)
- ISO 10993 Biological evaluation of medical devices (www.iso.org)
- WHO, International Clinical Trials Registry Platform (ICTRP) (http://www.who.int/ictrp/en/)

References

1. Wolf JS, Jr., Bennett CJ, Dmochowski RR, Hollenbeck BK, Pearle MS, Schaeffer AJ, et al. Best practice policy statement on urologic surgery antimicrobial prophylaxis. J Urol. 2008;179(4):1379-90.

2. Grabe M. Guidelines on urological infections. European Association of Urology. 2015.

3. Cai T, Verze P, Brugnolli A, Tiscione D, Luciani LG, Eccher C, et al. Adherence to European Association of Urology Guidelines on Prophylactic Antibiotics: An Important Step in Antimicrobial Stewardship. Eur Urol. 2016;69(2):276-83.

4. Krasnow RE, Mossanen M, Koo S, Kubiak DW, Preston MA, Chung BI, et al. Prophylactic Antibiotics and Postoperative Complications of Radical Cystectomy: A Population Based Analysis in the United States. J Urol. 2017.

5. Abe T, Takada N, Shinohara N, Matsumoto R, Murai S, Sazawa A, et al. Comparison of 90-day complications between ileal conduit and neobladder reconstruction after radical cystectomy: a retrospective multi-institutional study in Japan. Int J Urol. 2014;21(6):554-9.

6. Shabsigh A, Korets R, Vora KC, Brooks CM, Cronin AM, Savage C, et al. Defining early morbidity of radical cystectomy for patients with bladder cancer using a standardized reporting methodology. Eur Urol. 2009;55(1):164-74.

7. Lavallee LT, Schramm D, Witiuk K, Mallick R, Fergusson D, Morash C, et al. Peri-operative morbidity associated with radical cystectomy in a multicenter database of community and academic hospitals. PLoS One. 2014;9(10):e111281.

8. Gondo T, Ohno Y, Nakashima J, Hashimoto T, Takizawa I, Tanaka A, et al. Factors predicting incisional surgical site infection in patients undergoing open radical cystectomy for bladder cancer. Int J Clin Oncol. 2014;19(5):935-9.

9. Wuethrich PY, Burkhard FC, Thalmann GN, Stueber F, Studer UE. Restrictive deferred hydration combined with preemptive norepinephrine infusion during radical cystectomy reduces postoperative complications and hospitalization time: a randomized clinical trial. Anesthesiology. 2014;120(2):365-77.

10. Bootsma AM, Laguna Pes MP, Geerlings SE, Goossens A. Antibiotic prophylaxis in urologic procedures: a systematic review. Eur Urol. 2008;54(6):1270-86.

11. Grabe M, Botto H, Cek M, Tenke P, Wagenlehner FM, Naber KG, et al. Preoperative assessment of the patient and risk factors for infectious complications and tentative classification of surgical field contamination of urological procedures. World J Urol. 2012;30(1):39-50.

12. Matsumoto T, Kiyota H, Matsukawa M, Yasuda M, Arakawa S, Monden K, et al. Japanese guidelines for prevention of perioperative infections in urological field. Int J Urol. 2007;14(10):890-909.

13. Bratzler DW, Dellinger EP, Olsen KM, Perl TM, Auwaerter PG, Bolon MK, et al. Clinical practice guidelines for antimicrobial prophylaxis in surgery. Surg Infect (Larchmt). 2013;14(1):73-156.

14. Grabe M. Antibiotic prophylaxis in urological surgery, a European viewpoint. Int J Antimicrob Agents. 2011;38 Suppl:58-63.

15. Calvert JK, Holt SK, Mossanen M, James AC, Wright JL, Porter MP, et al. Use and outcomes of extended antibiotic prophylaxis in urological cancer surgery. J Urol. 2014;192(2):425-9.

16. Hara N, Kitamura Y, Saito T, Komatsubara S, Nishiyama T, Takahashi K. Perioperative antibiotics in radical cystectomy with ileal conduit urinary diversion: efficacy and risk of antimicrobial prophylaxis on the operation day alone. Int J Urol. 2008;15(6):511-5.

17. Macgowan AP, Surveillance BWPoR. Clinical implications of antimicrobial resistance for therapy. J Antimicrob Chemother. 2008;62 Suppl 2:ii105-14.

18. Ammerlaan HS, Harbarth S, Buiting AG, Crook DW, Fitzpatrick F, Hanberger H, et al. Secular trends in nosocomial bloodstream infections: antibiotic-resistant bacteria increase the total burden of infection. Clinical infectious diseases : an official publication of the Infectious Diseases Society of America. 2013;56(6):798-805.

19. Llor C, Bjerrum L. Antimicrobial resistance: risk associated with antibiotic overuse and initiatives to reduce the problem. Ther Adv Drug Saf. 2014;5(6):229-41.

20. Infections CNSDfSto. CDC/NHS Surveilance Definitions for Specific types of Infections. CDC Surveillance Definitions. 2017.

21. FDA. Guidance for Industry FDA Toxicity grading scale for healthy adults and adolescent volunteers enrolled in preventive vaccine trials. 2007.

17. APPENDICES

ICH: (NOTE: Since the protocol and the clinical trial/study report are closely related, further relevant information can be found in the ICH Guideline for Structure and Content of Clinical Study Reports.)

Please consider well with adding documents here that are very frequently changing, they may be mentioned as separately provided documents and listed here.

Except for medical devices, the section headings can be renamed accordingly.

1. IMP: IB or SPC

2. Medical Devices: IB (according to ISO 14155)

3. Medical Devices: Assurance of producer

4. Medical Devices: List of norms (vollständig eingehaltene, teilweise eingehaltene)

5. Radiolabelled products: Strahlenschutzverordnung

6. eg List of study sites / PIs

List of countries or centres where data will be collected or reference to where list of study sites can be obtained

7. Other

eg Specific protocols (e.g. MRI)

eg Case Report Form (e.g. CRF)

eg Patient Information and informed consent

Model of consent form and other related documentation given to participants and authorised surrogates and additional consent provisions for collection and use of participant data and biological specimens in ancillary studies, if applicable

eg Other material to patients

Model of consent form and other related documentation given to participants and authorised surrogates and additional consent provisions for collection and use of participant data and biological specimens in ancillary studies, if applicable
