# Supplementary material for: Perioperative Antibiotic Prophylaxis Duration in Patients Undergoing Cystectomy With Urinary Diversion: A Randomized Clinical Trial
Source: JAMA Netw Open. 2024 Oct 18;7(10):e2439382. doi: 10.1001/jamanetworkopen.2024.39382 (PMC11581670; doi:10.1001/jamanetworkopen.2024.39382)
Supplement: Supplement 2. — eTable 1. Antibiotic Regimen Used for Perioperative Prophylaxis eTable 2. Perioperative Care eTable 3. Diagnosis of Surgical Site Infections (e CRF Form) eTable 4. Intention-to-Treat (ITT) Sensitivity Analysis eTable 5. Cumulative Incidences for Surgical Site Infection and Death [file jamanetwopen-e2439382-s002.pdf]

Supplemental Online Content

Thurnheer MC, Schürmann A, Huber M, Marschall J, Wuethrich PY, Burkhard FC. Perioperative antibiotic prophylaxis duration in patients undergoing cystectomy with urinary diversion: a randomized clinical trial. *JAMA Netw Open*. 2024;7(10):e2439382. doi:10.1001/jamanetworkopen.2024.39382

eTable 1. Antibiotic Regimen Used for Perioperative Prophylaxis

eTable 2. Perioperative Care

eTable 3. Diagnosis of Surgical Site Infections (e CRF Form)

eTable 4. Intention-to-Treat (ITT) Sensitivity Analysis

eTable 5. Cumulative Incidences for Surgical Site Infection and Death

This supplemental material has been provided by the authors to give readers additional information about their work.

© 2024 Thurnheer MC et al. *JAMA Network Open.*

eTable 1. Antibiotic Regimen Used for Perioperative Prophylaxis

• Patients in the 24h PAP group received tobramycin loading dose preoperatively 120 mg then 3 x 80 mg. or adapted to creatinine clearance for max. 24h, metronidazole: loading dose preoperatively 1000 mg, then 2 x 500 mg i.v. for max. 24h and amoxicillin/clavulanate loading dose preoperatively 1.2 g then 3 x 1.2 g/d i.v. for max. 24h.
• Patients in the extended PAP group received tobramycin loading dose preoperatively 120 mg then 3 x 80 mg i.v. (or adapted to creatinine clearance) for 48h, metronidazole loading dose preoperatively 1000 mg, then 2 x 500 mg i.v. for 48h and amoxicillin/clavulanate loading dose preoperatively 1.2 g then 3 x 1.2 g/d i.v. until all catheter/stents/drains removed.
• In patients allergic to penicillin or beta lactam antibiotics, amoxicillin/clavulanate was replaced by a combination of vancomycin (2x1 g/24h, or adapted to creatinine clearance) and ciprofloxacin 500 mg/d according to current standard practice. The first dose of antibiotics was administered within 1 hour before skin incision. PAP was stopped 24h post-surgery in the 24h PAP group and after removal of all catheters/stents (>48h post-surgery) in the extended PAP group.
**Antibiotics during removal of transurethral catheter**
• In patients with orthotopic bladder substitutes in the long arm antibiotics were stopped 2 days before removal of urethral catheter for 48h. After that urine culture was the obtained and the patient received trimethoprim/sulfamethoxazole for 5-7 days. In the short am trimethoprim/sulfamethoxazole was initiated when the transurethral catheter was removed (standard practice).

© 2024 Thurnheer MC et al. *JAMA Network Open.*

eTable 2. Perioperative Care

Standardized perioperative care was used to facilitate postoperative recovery

Preoperative:

- Protein enriched nutrition
- Active life style stimulation
- No enteral bowel preparation
- Normal nutrition till midnight before surgery
- Clear drinks including carbohydrate loading till 2 h before surgery
- Subcutaneous injection of low molecular heparin at 20:00 p.m.
- Deep vein thrombosis (DVT) prophylaxis with TED hose and sequential compression devices

Intraoperative:

- Restrictive intravenous fluid regimen aiming a zero postoperative weight gain
- Gastrostomy tube placed, removal of orogastric tube at end of procedure
- Opioid sparing analgesia strategy using regional anesthesia (epidural or TAP blocks), and intravenous administration of clonidine or continuous intravenous administration of dexmedetomidine.
- Postoperative nausea and vomiting (PONV) prophylaxis intravenous administration of mephameson 8 mg during induction of anesthesia and ondansetron 4 mg intravenously at the end of the procedure.

Postoperative:

- DVT prophylaxis with ambulation, TED, and subcutaneous low molecular heparin (weight adapted), started 6 hours postoperatively
- Chewing gum encouraged
- Clear drinks allowed the same evening after surgery
- Gastrostomy tube initially left on drainage, closure of the gastrostomy tube without nausea and vomiting for >24 h
- Bedside mobilization as soon as possible, ideally the same evening after surgery, if not possible not later than the next morning
- Initial pain treatment with thoracic epidural analgesia, no opioids
- Ambulation and spending time in the chair on POD 1
- Start oral fluids including energy protein based drinks on POD 1
- Unrestricted clear drinks on POD 1
- Prokinetics: start with 0.25mg neostigmin s.c. up to 2 times per day on POD 2, after that increase to max. 4 times per day.
- Small snacks introduced on POD 2, not later than POD 3
- POD 3: Encourage longer mobilization, walking distance and spending time in the chair
- Antiemetics only given on request
- Drains removed if draining <50ml/day
- Gastrostomy tube removed once the patient passed stool
- POD 5: thoracic epidural removed, oral analgesics (Metamizol, Paracetamol, Hydroxycodon/Naloxon on demand)

© 2024 Thurnheer MC et al. *JAMA Network Open.*

eTable 3. Diagnosis of Surgical Site Infections (e CRF Form)

186	[date_ssi_diag]	date of SSi Diagnosis	text
187	[ssi_any]	Any signs of surgical site infections?	yesno<br>1 Yes<br>0 No
188	[superficial_ssi]<br>Show the field ONLY if:<br>[ssi_any] = '1'	Superficial SSI<br>at least one of the following:	checkbox, Required<br>1 superficial_ssi___1 purulent discharge<br>2 superficial_ssi___2 microorganism identified from surgical site<br>3 superficial_ssi___3 superficial incision for purpose of drainage<br>4 superficial_ssi___4 superficial SSI clinically diagosed by surgeon/attending physician<br>5 superficial_ssi___5 none of the above
189	[superficialssiyn]<br>Show the field ONLY if:<br>[ssi_any] = '1'	Superficial SSI<br>yes/no	yesno, Required<br>1 Yes<br>0 No
190	[deep_ssi]<br>Show the field ONLY if:<br>[ssi_any] = '1'	Deep incisional SSI<br>at least one of the following:	checkbox, Required<br>1 deep_ssi___1 purulent drainage from deep incision<br>2 deep_ssi___2 spontaneous or deliberate opening of deep incision and microorganism identified<br>3 deep_ssi___3 spontaneous or deliberate opening of deep incision AND pain/tenderness/T>38°C<br>4 deep_ssi___4 abscess or other evidence of deep incision infection detected on gross anatomical exam or imaging test<br>5 deep_ssi___5 none of the above
191	[deepincisionalssiyn]<br>Show the field ONLY if:<br>[ssi_any] = '1'	Deep incisional SSI<br>yes/no	yesno, Required<br>1 Yes<br>0 No
192	[organ_space_ssi]<br>Show the field ONLY if:<br>[ssi_any] = '1'	Organ/Space SSI<br>at leat one of the following:	checkbox, Required<br>1 organ_space_ssi___1 purulent drainage from drain placed into organ/space<br>2 organ_space_ssi___2 organism identified in specimen obtained from the organ/space<br>3 organ_space_ssi___3 abscess or other evidence of deep organ/space infection on anatomical exam or imaging test<br>4 organ_space_ssi___4 none of the above
193	[organ_spacessiyn]<br>Show the field ONLY if:<br>[ssi_any] = '1'	Organ/Space SSI<br>yes/no	yesno, Required<br>1 Yes<br>0 No

© 2024 Thurnheer MC et al. *JAMA Network Open.*

eTable 4. Intention-to-Treat (ITT) Sensitivity Analysis

(A) Missing outcomes of 6 excluded patients imputed with no event (neither SSI nor death)

	24h PAP N=100	Extended PAP N=99	p-value
**Any SSI during follow-up** [N (%, 95%-CI)]	8 (8.0%, 95%-CI: 3.8% - 15.6%)	12 (12.1%, 95%-CI: 6.7% - 20.6%)	0.46

Competing risk regression		Outcome: SSI		Outcome: Death
**Treatment group:**	**N**	**Hazard ratio (HR, 95%-CI)**	**N**	**Hazard ratio (HR, 95%-CI)**
Extended PAP	99	ref.	99	ref.
24h PAP	100	0.65 (95%-CI: 0.27 – 1.57, p = 0.34)	100	1.24 (95%-CI: 0.34 – 4.56, p = 0.75)

Primary outcome: SSI		
**Risk difference** (90-day cumulative incidence)		**Non-inferiority analysis** (margin: 10%)
24h PAP – Extended PAP	-4.1% (90%-CI: -11.1% – 2.9%, 95%-CI: -12.6 – 4.3%)	established

(B) Missing outcomes of 6 excluded patients imputed with SSI event at day 10 (no death)

	24h PAP N=100	Extended PAP N=99	p-value
**Any SSI during follow-up** [N (%, 95%-CI)]	13 (13.0%, 95%-CI: 7.4% - 21.6%)	13 (13.1%, 95%-CI: 7.5% - 21.8%)	0.09

Competing risk regression		Outcome: SSI		Outcome: Death
**Treatment group:**	**N**	**Hazard ratio (HR, 95%-CI)**	**N**	**Hazard ratio (HR, 95%-CI)**
Extended PAP	99	ref.	99	ref.
24h PAP	100	1.00 (95%-CI: 0.47 – 2.11, p>0.99)	100	1.24 (95%-CI: 0.34 – 4.56, p = 0.75)

Primary outcome: SSI		
**Risk difference** (90-day cumulative incidence)		**Non-inferiority analysis** (margin: 10%)
24h PAP – Extended PAP	-0.2% (90%-CI: -8.1% – 7.7%, 95%-CI: -9.6 – 9.2%)	established

© 2024 Thurnheer MC et al. *JAMA Network Open.*

eTable 5. Cumulative Incidences for Surgical Site Infection and Death

Cumulative incidences (competing risk)				
	Outcome SSI		*Outcome death*	
**Follow-up time:**	**24h PAP**	**Extended PAP**	**24h PAP**	**Extended PAP**
7 days	2.1% (95%-CI: 0.4% - 6.7%)	2.0% (95%-CI: 0.4% - 6.5%)	1.1% (95%-CI: 0.1% - 5.2%)	0% (95%-CI: NA)
10 days	4.2% (95%-CI: 1.4% - 9.7%)	6.1% (95%-CI: 2.5% - 12.1%)	1.1% (95%-CI: 0.1% - 5.2%)	1.0% (95%-CI: 0.1% - 5.0%)
30 days	8.4% (95%-CI: 3.9% - 15.1%)	12.2% (95%-CI: 6.7% - 19.6%)	2.1% (95%-CI: 0.4% - 6.7%)	2.0% (95%-CI: 0.4% - 6.5%)
90 days	8.4% (95%-CI: 3.9% - 15.1%)	12.2% (95%-CI: 6.7% - 19.6%)	5.3% (95%-CI: 1.9% - 11.1%)	4.1% (95%-CI: 1.3% to 9.4%)
Gray's Test	0.39		0.70	

© 2024 Thurnheer MC et al. *JAMA Network Open.*
