# Supplementary material for: Perioperative Antibiotic Prophylaxis Duration in Patients Undergoing Cystectomy With Urinary Diversion: A Randomized Clinical Trial
Source: JAMA Netw Open. 2024 Oct 18;7(10):e2439382. doi: 10.1001/jamanetworkopen.2024.39382 (PMC11581670; doi:10.1001/jamanetworkopen.2024.39382)
Supplement: Supplement 3. — Data Sharing Statement [file jamanetwopen-e2439382-s003.pdf]

Data Sharing Statement

Thurnheer. Perioperative Antibiotic Prophylaxis Duration in Patients Undergoing Cystectomy With Urinary Diversion. *JAMA Netw Open*. Published October 18, 2024. doi:10.1001/jamanetworkopen.2024.39382

Data
Additional Information: ClinicalTrials.gov https://clinicaltrials.gov/study/NCT03305627?tab=results NCT03305627
Data available: No

Additional Information
Explanation for why data not available: Data will be made available upon specific request
